# Specific splice junction detection in single cells with SICILIAN

**DOI:** 10.1186/s13059-021-02434-8

**Published:** 2021-08-05

**Authors:** Roozbeh Dehghannasiri, Julia Eve Olivieri, Ana Damljanovic, Julia Salzman

**Affiliations:** 1 Department of Biochemistry, Stanford University, Stanford, CA 94305 USA; 2 Institute for Computational and Mathematical Engineering, Stanford University, Stanford, CA 94305 USA; 3 Seven Bridges Genomics Inc., Cambridge, MA 02142 USA; 4 Department of Biomedical Data Science, Stanford University, Stanford, CA 94305 USA

## Abstract

Precise splice junction calls are currently unavailable in scRNA-seq pipelines such as the 10x Chromium platform but are critical for understanding single-cell biology. Here, we introduce SICILIAN, a new method that assigns statistical confidence to splice junctions from a spliced aligner to improve precision. SICILIAN is a general method that can be applied to bulk or single-cell data, but has particular utility for single-cell analysis due to that data’s unique challenges and opportunities for discovery. SICILIAN’s precise splice detection achieves high accuracy on simulated data, improves concordance between matched single-cell and bulk datasets, and increases agreement between biological replicates. SICILIAN detects unannotated splicing in single cells, enabling the discovery of novel splicing regulation through single-cell analysis workflows.

**Supplementary Information:**

The online version contains supplementary material available at 10.1186/s13059-021-02434-8.

## Main text

Alternative splicing is essential for the specialized functions of eukaryotic cells, necessary for development [1], and a greater contributor to genetic disease burden than mutations [2]. Despite the importance of splicing and massive single-cell RNA-seq (scRNA-seq) data generated, the extent to which the diversity of RNA splicing in single cells is regulated and functional versus transcriptional noise remains contentious [3].

**Fig. 1:**
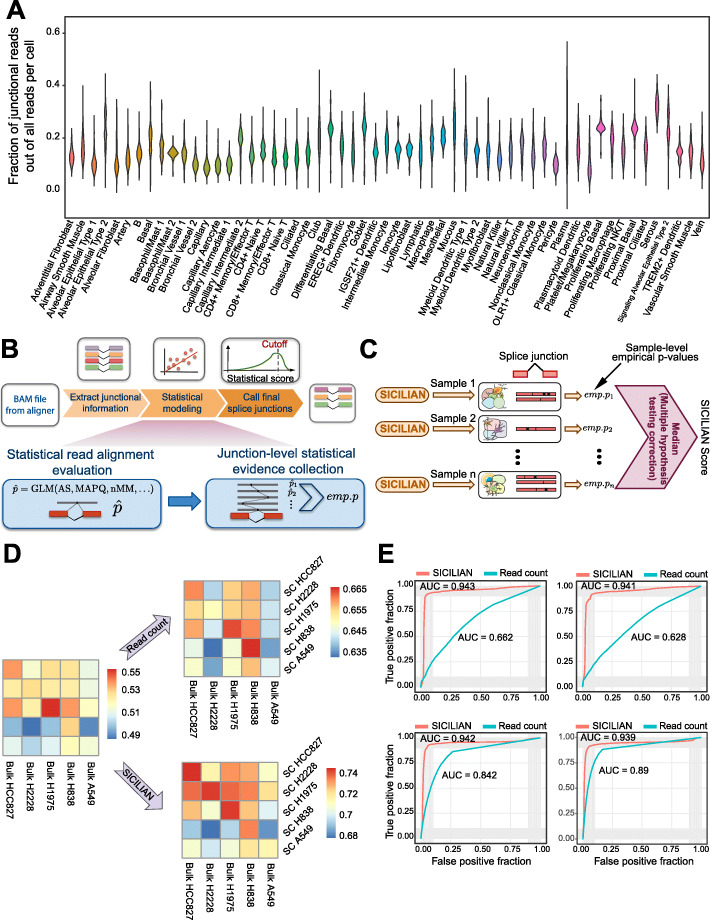
Overview of the SICILIAN statistical framework and its performance evaluation based on benchmarking datasets. **A** High and variable fraction of junctional reads across diverse cell types in the HLCA dataset [11]. Each violin plot shows the fraction of mapped reads in each cell (within a cell type) that are junctional. **B** SICILIAN takes the alignment information file (usually in the form of a BAM file) from a spliced aligner such as STAR and then deploys its statistical modeling to assign a statistical score to each junction. **C** SICILIAN utilizes the cell-level statistical scores (empirical *p* values) for each junction across 10x samples to correct for increased false discovery rates due to multiple hypothesis testing. The corrected score is called the “SICILIAN score” and can be used to consistently call junctions across cells. (D) SICILIAN improves the concordance between detected splicing junctions in single cells and bulk cell lines. (E) ROC curves by SICILIAN and read count criteria for four simulated datasets [8, 12] (the top two based on data from [12] and the bottom two based on data from [8])

Current spliced aligners call many false-positive spliced junctions, partly because they are computational procedures operating on noisy observations of expressed RNA. The factors influencing this noise include sequence properties of the genome: repetitive genomic sequence within and between genes, biochemical noise introduced during library preparation which could cause mismatches, template switches, and technical noise causing base call errors during sequencing [4]. Differences between the reference and sequenced transcriptomes caused by polymorphisms and other genetic variations yield further false positives during the process of alignment [5–8]. False-positive alignments are further exacerbated in scRNA-seq due to higher levels of biochemical noise specific to single-cell preparations (i.e., higher prevalence of low-entropy-reads in scRNA-Seq as discussed below) as well as statistical issues: false-positive splice junction calls are more prevalent in larger datasets. Together, these challenges combine to generate an ongoing debate regarding whether 10x Chromium (10x) can be used for reliable de novo splice junction detection [9, 10], despite the presence of a large number of junctional reads in the datasets generated by this protocol (Fig. 1A).

The resolution and massive number of available scRNA-seq datasets hold great promise for discovering regulatory and functional splicing biology, including (a) identifying novel unannotated regulated alternative splicing in rare cell types present even in well-annotated organisms such as human [13], (b) de novo prediction of dysregulated splicing in single-cell data from diseases such as in cancer or neurodegenerative diseases, and (c) automatic high-quality junction prediction from poorly annotated organisms such as new model organisms.

Typical workflows from commodity platforms such as 10x genomics (e.g., Cell Ranger [14]) have preprocessing steps that remove all unannotated junctions before reporting spliced alignments. Further, the vast majority of publications characterizing splicing in single cells require ad hoc lower bounds on junction overlap or total number of supporting reads [15]. These approaches will miss and highlight the need for a statistically driven method to discover novel regulation of splicing in single cells. There is thus a great biological need for a method that both reduces the false-positive identification of junctions while having power to detect bona fide unannotated splicing (controlling false negatives). Overall, existing approaches to splicing analysis in the scRNA-seq data either lack sufficient sensitivity to identify splice junctions or specificity to identify false positives [8].

In this paper, we introduce SICILIAN (SIngle Cell precIse spLice estImAtioN), a statistical wrapper for precise splice junction quantification in single cells. SICILIAN deconvolves biochemical noise (generated during library preparation) and computational noise (generated by the spliced aligner while mapping reads to the genome), both being highly prevalent in scRNA-seq and can lead to false-positive junctions. To identify spliced alignments that are erroneously reported by the aligner due to this combined noise, SICILIAN employs generalized linear statistical modeling, with predictors being various read mapping features. In this paper, we use STAR [16] as the spliced aligner in SICILIAN, though the general statistical framework can be applied to refine the splice junction calls from any spliced aligner generating a BAM file. SICILIAN can be applied to both single-end (such as 10x) and paired-end data (such as Smart-seq 2). When running on paired-end data, SICILIAN utilizes alignment features for both mate reads (R1 and R2) in its model and extracts spliced junctions from both R1 and R2 alignments.

The SICILIAN algorithm has three main steps: (1) assign a statistical score to each junctional read’s alignment to quantify the likelihood that the read alignment is truly from RNA expression rather than artifacts; (2) aggregate read scores to summarize the likelihood that a given junction is a true positive; and (3) report single-cell resolved junction expression quantification, corrected for multiple hypotheses testing ( “Methods” section, Fig. 1B,C).

The goal of step (1) above is to statistically evaluate the confidence of the alignment for each junctional read. To do this, SICILIAN fits a penalized generalized linear model [17] on the input RNA-seq data, where positive and negative training classes are defined based on whether each junctional read also has a genomic alignment. Our analysis has shown that this definition for training data well approximates the alignment profile of reads aligned to the false-positive and true-positive junctions (Additional file 1: Fig. S1, Fig. S2). Furthermore, training a new model for each input dataset allows SICILIAN to adapt to batch effects. The model uses the following predictors: the number of alignments for the read, the number of bases in the longer and shorter read overhangs on each side of the junction, the alignment score adjusted by the read length*,* the number of mismatches, the number of soft-clipped bases, and read entropy ( “Methods” section). These predictors have the power to distinguish reads aligned to false-positive and true-positive junctions defined by ground truth from simulated datasets. The positive and negative training reads used for training the model can reliably model the general profile of the true-positive and false-positive reads (Additional file 1: Fig. S1, Fig. S2).

Read entropy, a quantitative measure of how repetitive a sequence is, is not generally appreciated as an important variable in scRNA-seq reads even though it is characteristic of technical artifacts [18], underlining its importance in the SICILIAN model. Read sequence entropy is expected to be a highly informative predictor of false-positive spliced alignments for two reasons: (1) reverse transcriptase or PCR enzymes are known to generate sequences of low entropy (“PCR stutter”) [18], and PCR crossover is common in these regions; (2) these low-entropy sequences typically map to many places in the genome [19]. Also, the entropy could be more informative and variable in scRNA-seq compared to bulk RNA-seq. For example, in the 10x data from a human lung study [11] (HLCA data set), the average read entropy in 20% of cells is < 4 (Additional file 1: Fig. S3A), which is much more than the fraction of low-entropy reads in bulk RNA-seq datasets, where the entropy is < 4 in only 0.09% and 0.4% of reads in one simulated^8^ and five bulk cell lines, respectively (Additional file 1: Fig. S3B,C).

In step (2), the statistical scores assigned to each junction’s aligned reads are aggregated using a Bayesian hypothesis testing framework to obtain aggregated junction-level scores. SICILIAN subsequently uses the distribution of aggregated scores for likely false-positive junctions to predict an empirical *p* value for each junction. Finally, in step (3), SICILIAN corrects for multiple hypotheses testing by taking the median of the empirical p-values for each junction across samples and reports it as the final “SICILIAN score” for the junction (Fig. 1C). User-defined thresholding on this score allows for a junction to be either called or thrown out consistently across all samples. In this paper, we used a threshold of 0.15, which was selected to maximize the sum of sensitivity and specificity on the benchmarking datasets with known ground truth (“Methods” section).

We benchmarked SICILIAN using two different types of benchmarking data: matched scRNA-seq and bulk data from five human lung adenocarcinoma cell lines [20] and simulated data with known ground truth [8, 12]. We compared SICILIAN to commonly used filtering criteria in the field: all junction calls based on STAR [16] raw alignments, the junctions supported only by uniquely mapping reads [21], and calling junctions based on read counts [22, 23].

SICILIAN increases the concordance of junction calls on matched single-cell and bulk datasets [20] (Fig. 1D; Additional file 1: Fig. S4A). We define “concordance” to be the fraction of junctions detected in the single cells from a cell line that are also present in the bulk data from that cell line. SICILIAN increases the concordance between the detected junctions from 10x and bulk RNA-seq regardless of the pairs’ cells of origin, which is consistent with SICILIAN identifying and removing scRNA-seq-specific artifacts (i.e., false-positive junctions that are present in only scRNA-Seq data). SICILIAN improves the concordance for all cell lines (Fig. 1D), e.g., for cell line HCC827, the concordance based on raw STAR calls is 0.54, and SICILIAN increases it to 0.75, while calling junctions based on a 10-read filter only increases the concordance to 0.66. Also, considering junctions detected in the single-cell and bulk datasets of the same cell line as true positives, SICILIAN outperforms the read count criterion in terms of the AUC value (Additional file 1: Fig. S4B).

As there is no single-cell dataset with fully-known ground truth and SICILIAN’s modeling is general and can be applied to both bulk and scRNA-Seq, we resorted to bulk-level simulated datasets and ran the identical SICILIAN model on them. SICILIAN increases prediction accuracy on four bulk simulated datasets with known ground truth [8, 12]. For these datasets, SICILIAN uniformly achieves AUCs of ~ 0.94, a significant increase over the AUCs of 0.66–0.89 based on the read count criterion (Fig. 1E).

**Fig. 2:**
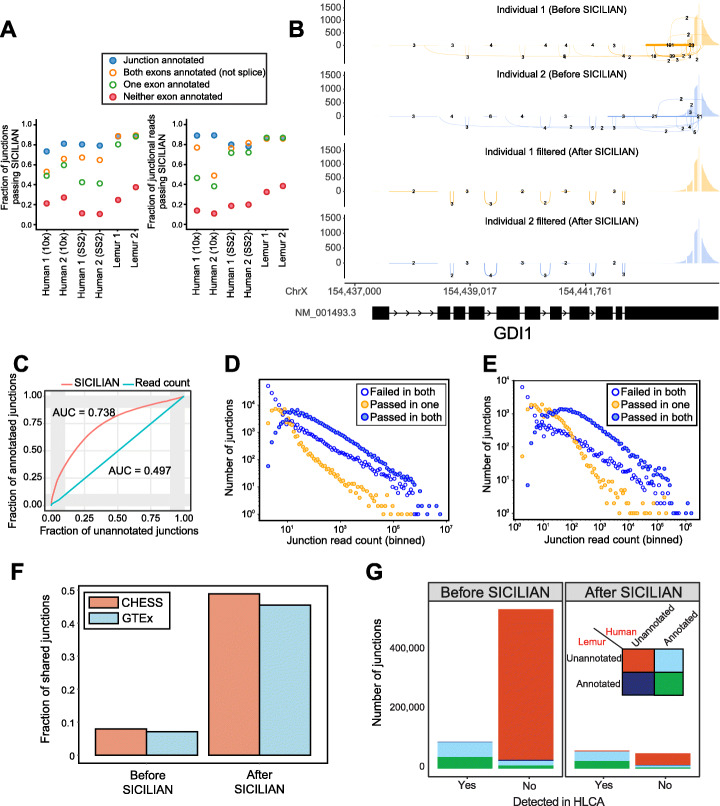
Splice junction discovery in human lung (HLCA) and mouse lemur lung (MLCA) cells. **A** SICILIAN filters out a higher proportion of unannotated junctions than annotated in all individuals from both human and mouse Lemur datasets [only junctions with at least two reads in the given dataset are plotted]. **B** Splice junctions identified by SICILIAN in gene *GDI1* in human; annotated splicing was maintained and unannotated noisy junctions were all removed. **C** Better discrimination between annotated and unannotated junctions in the HLCA dataset achieved by the SICILIAN statistical criterion. **D** The number of junctions found in both human individuals that are called consistently by SICILIAN is larger than the number that are called differently in almost every case, regardless of the number of junctional reads. **E** The number of junctions called consistently between the two mouse lemur individuals is also larger than the number called inconsistently at almost every read depth after SICILIAN filtering. **F** The fraction of HLCA junctions that are found in CHESS and GTEx databases before and after applying SICILIAN to STAR raw calls. **G** The number of mouse lemur junctions orthologous junctions (found by LiftOver from Mmur3 to hg38) that have been also detected in the HLCA dataset. Junctions have been further classified based on their annotation status in the mouse lemur and human transcriptomes

In addition to benchmarking datasets, we ran SICILIAN on 36,583 10x and 6,565 Smart-seq2 (SS2) cells from two individuals from the human lung cell atlas (HLCA) [11] and 16,755 10x lung cells from two individuals from the mouse lemur cell atlas (MLCA) [24]. Since there is no ground truth for real data, we use annotation status as an approximate surrogate for ground truth. Knowing that many annotated junctions have been manually curated or experimentally validated, we expect that an algorithm with ability to correctly identify false positives should enrich for annotated junctions, particularly for organisms with extensive annotation such as humans [25]. Because transcript annotations are not part of the SICILIAN model, this serves as an orthogonal measure for performance evaluation. SICILIAN modeling increases the proportion of annotated to unannotated junctions in all four human and mouse lemur individuals compared to the original STAR calls and read-count criterion (Fig. 2). In all 10x datasets from four human and mouse individuals, SICILIAN calls a higher proportion of annotated junctions (83.6% of annotated junctions are called on average out of all annotated junctions) than unannotated junctions (29.2% of unannotated junctions are called on average out of all unannotated junctions), and a higher proportion of annotated junctional reads (87.6% of annotated junctional reads are called on average out of all annotated junctional reads) than unannotated junctional reads (23.9% of unannotated junctional reads are called on average out of all unannotated junctional reads), excluding junctions that only appear once in the dataset (Fig. 2A; Additional file 1: Fig. S5). For many genes, including *GDI1*, *GOT2*, and *CD14*, SICILIAN removed all noisy unannotated junctions and kept only annotated junctions although the algorithm was agnostic to the annotation (Fig. 2B; Additional file 1: Fig. S6). Considering annotated and unannotated junctions as surrogates for true-positive and false-positive junctions in human lung data, SICILIAN achieves an AUC of 0.74, while that of the read-count-based approach is 0.5 (Fig. 2C). Note that due to the power of scRNA-seq in capturing cells from rare cell types, if there are unannotated junctions with low read counts in these rare cell types, we would not be able to detect those junctions based on a read-count criterion and need a better detection regime such as SICILIAN for calling them.

SICILIAN also increases the agreement of splicing calls between individuals. At almost all fixed junction expression levels, SICILIAN makes more consistent decisions (either calls or rejects the junction in both individuals) than inconsistent decisions for both human and mouse lemur, emphasizing the robustness of SICILIAN (Fig. 2D,E). It calls 117,684 shared junctions between individuals in HLCA 10x, while a 10-read cutoff calls only 80,292 (Fig. 2D). SICILIAN makes a consistent decision for 83.0% of junctions. Similarly, in MLCA, SICILIAN calls 36,446 shared junctions in both individuals, much higher than 17,798 that were called by a 10-read cutoff criterion, and makes a consistent decision about 69.9% of junctions.

To further identify whether SICILIAN enriches for known junctions, we compared the junctions called by SICILIAN in HLCA 10x with two of the most recent and precise databases of human splice junctions: CHESS [25] and the Genotype-Tissue Expression (GTEx) project [26]. Only 8% and 7% of raw STAR calls are present in CHESS and GTEx, respectively, but SICILIAN increases these percentages to 48.8% and 45.4%, respectively (Fig. 2F). We also looked at the junctions within each lung cell type and found that the fraction of junctions that are not present in CHESS or GTEx varies substantially across different cell types, even those with similar sequencing depth, indicating that the rate of novel splicing may vary between cell types (Additional file 1: Fig. S7).

Finally, mouse lemur calls by SICILIAN are enriched for having annotated orthologous junctions (obtained via the LiftOver tool [27]) in the human transcriptome, which is much more complete than the mouse lemur transcriptome [28] (“Methods” section). Strikingly, more than 48.4% of lemur-unannotated junctions called by SICILIAN are annotated in the human transcriptome, compared to only 11.2% of unfiltered STAR calls, which supports the claim that SICILIAN filtering enriches for true-positive junctions and highlights the power of SICILIAN for improving transcriptome annotation in poorly studied organisms. We also compared the detected junctions in MLCA and HLCA datasets: applying SICILIAN increases the fraction of junctions in mouse lemur that have been also detected in HLCA from 15 to 54% (Fig. 2G).

Taken together, our results demonstrate that the SICILIAN method enables a new level of precision in splice junction detection from single-cell platforms such as 10x and SS2. SICILIAN allows automatic junction discovery even for poorly annotated splicing programs such as rare cell types or in new model organisms. The conceptual models used in SICILIAN are also applicable to other data types such as emerging single-cell sequencing technologies and bulk sequencing, exemplified by the recent application of SICILIAN for detecting SARS-COV-2 subgenomic RNAs in the swab samples taken from patients [19]. Additionally, we anticipate that this framework can be expanded to detect nonlinear RNA variants such as gene fusions and circRNAs using chimeric alignments reported by the aligner. Underlining the importance of unannotated junction discovery, SICILIAN discovered new regulated splicing patterns in primary human samples that were impossible using annotations [29].

## Methods

### Alignment of scRNA-seq data

FASTQ files were aligned using STAR [16] (v 2.7.5.a) with default parameters except for chimSegmentMin = 10 and chimJunctionOverhangMin = 10. Every non-chimeric spliced alignment (defined as a read with an “N” in its CIGAR string) was parsed from the STAR BAM file. By collapsing spliced alignments based on their mapping positions, we obtained the “unfiltered STAR calls.” If a read had multiple spliced alignments, we only included the spliced alignment with the lowest value of the HI BAM flag to avoid double-counting reads. We also kept track of which reads had genomic alignments as needed for selecting our training reads in statistical modeling. When input data is paired-end, both BAM files corresponding to R1 and R2 reads were parsed for extracting spliced alignments.

### Statistical detection of splice junctions in single-cell data

SICILIAN extracts all relevant information for the spliced alignments from the BAM file and utilizes that information to build a statistical model to distinguish truly expressed junctions from false positives due to biochemical and computational noise. The statistical framework in SICILIAN can be divided into three main steps: (1) statistical read alignment evaluation, (2) junction-level statistical evidence collection, and (3) multiple hypothesis testing correction (Fig. 1B). To build the model, SICILIAN takes advantage of the information across thousands of likely false-positive and true-positive read alignments to train a logistic regression model and thereby assigns a single statistical score to each extracted spliced alignment. Therefore, for each sample that SICILIAN is run on, a new model is built, allowing the model to specifically adapt to the experimental conditions of the given sequencing run. For the data analyzed in this paper, each 10x lane (and SS2 cell) was modeled separately to allow the modeling of lane-specific batch effects.

### Statistical read alignment evaluation

To train the regression model, we define negative training reads as the junctional reads with genomic (i.e., a contiguous mapping to a genomic region) alignment and positive training reads as the junctional reads without a genomic alignment. Our choice of negative training reads reflects the mapping profile of erroneous alignments as the vast majority of junctional reads with genomic alignments are not due to the real splice junction expression, but rather confounding factors such as genome homology and other biochemical and sequencing errors. Our analysis on simulated data with known ground truth has shown that the mapping profiles of negative and positive reads are highly similar to those of true-positive and false-positive junctional reads (Additional file 1: Fig. S1, Fig. S2). Another advantage is that our selection criterion for training reads is independent of the predictors in the regression (because whether a spliced read also has a genomic alignment is not included as a feature in the model). Therefore, the training reads would not give too much weight to any predictor in the fitted regression model. Each positive and negative training set can have at most 10,000 junctional reads, chosen randomly among the set of reads satisfying the training reads criterion.

Let *y* be a binary variable, where *y* = 1 indicates a true spliced alignment and *y* = 0 indicates a false alignment. We model *p* (*y* = 1), the likelihood of a true spliced alignment, with the logistic regression with penalized maximum likelihood [17]. The predictors in the regression comprise: number of reported alignments for the read obtained from the NH tag in the BAM file (NH), number of mismatches obtained from the NM tag in BAM file (nmm), length-adjusted alignment score obtained by normalizing the alignment score from the AS tag to the read length (AS), length of the shorter read overhang flanking the junction (overlap), length of the longer read overhang flanking the junction (max_overlap), number of the soft-clipped bases given by the S segment of the CIGAR string (*S*), and entropy of the read sequence (entropy):

$$ {y}_i\sim \mathrm{GLMnet}\left(\mathrm{AS}+\mathrm{NH}+\mathrm{nmm}+\mathrm{overlap}\times \max \_\mathrm{overlap}+S+\mathrm{entropy}\right) $$

When processing paired-end data (such as SS2), we utilize the same alignment features in the model but now from both R1 and R2 reads:

$$ {y}_i\sim \mathrm{GLMnet}\ \left({\mathrm{AS}}_{\mathrm{R}1}+{\mathrm{AS}}_{\mathrm{R}2}+{\mathrm{NH}}_{\mathrm{R}1}+{\mathrm{NH}}_{\mathrm{R}2}+{\mathrm{nmm}}_{\mathrm{R}1}+{\mathrm{nmm}}_{\mathrm{R}2}+{\mathrm{overlap}}_{\mathrm{R}1}\times \max \_{\mathrm{overlap}}_{\mathrm{R}1}+{\mathrm{overlap}}_{\mathrm{R}2}\times \max \_{\mathrm{overlap}}_{\mathrm{R}2}+{\mathrm{S}}_{\mathrm{R}1}+{\mathrm{S}}_{\mathrm{R}2}+{\mathrm{entropy}}_{\mathrm{R}1}+{\mathrm{entropy}}_{\mathrm{R}2}+\mathrm{location}\_\mathrm{compatible}+\mathrm{strand}\_\mathrm{compatible}\right) $$

Note that we use location_compatible and strand_compatible as binary predictors for paired-end data to determine whether the mate reads are compatible with being generated from the same cDNA fragment, given that in paired-end reads one mate (R1) should map to the forward strand and the other mate (R2) should map to the reverse strand. By adopting this regression model, SICILIAN evaluates spliced alignments for all cells within a 10x sample, providing enough statistical evidence for identifying false-positive and true-positive junctions even in the cells with low read coverage. We fitted the regression using the GLMnet R package [17]. The fitted model is then applied to each junctional read to estimate a read-level score *p̂*, the estimated likelihood that the spliced alignment is true.

### Junction-level statistical evidence collection

The list of extracted junctions from the BAM file with their aligned reads is obtained by collapsing the spliced alignments based on their mapping position. For each junction with N aligned reads, the read-level scores are aggregated under a Bayesian hypothesis testing framework to obtain an aggregated junction-level score:

$$ P=\frac{\Pi_{i\in N}{\hat{p}}_i}{\Pi_{i\in N}{\hat{p}}_i+{\Pi}_{i\in N}\left(1-{\hat{p}}_i\right)}=\frac{1}{1+\exp\ {\sum}_{i\in N}\log\ \left(\frac{1-{\hat{p}}_i}{{\hat{p}}_i}\right)} $$

Since read-level scores are always between 0 and 1 and they are multiplied in the aggregated score, the aggregated score is biased against junctions with more reads even when their read alignments have high confidence. To correct for this bias, for each number N of aligned reads, we build a null distribution of aggregated scores by randomly sampling N reads across all read alignments. We then compute a junction cumulative score *P*_*cum*_ for each aggregated score *P* by comparing it against the null distribution. For junctions with *N* < 15 reads, we build the empirical null distribution by computing 10,000 random aggregated scores; for junctions with *N* > 15 reads, we use the central limit theorem to model the null distribution as a Gaussian distribution and use it to compute the cumulative score *P*_*cum*_.

To systematically estimating the SICILIAN’s false discovery rate, we further compute an empirical *p* value (*emp.p*) for each junction. To do so, we use the distribution of the cumulative scores *P*_*cum*_ for the junctions with at least 10% of their aligned reads having genomic alignment as well. Since a spliced read with a genomic alignment is likely a false positive due to artifacts, we use the splice junctions with a considerable fraction of aligned reads with genomic alignment as likely artifactual junctions and use the distribution of their cumulative scores *P*_*cum*_ as the null distribution to compute *emp.p*’s for other junctions. When SICILIAN is applied to a single sample (one 10x sample or one SS2 cell), *emp.p* is the SICILIAN’s final score for the junctions in the sample and junctions with *emp.p* less than a certain threshold (e.g., 0.1) are called by SICILIAN.

### Multiple hypothesis testing correction

When analyzing multiple 10x samples or SS2 cells (which is typical in single-cell studies), merely using empirical p-values for detecting junctions might result in an increased false discovery rate due to multiple hypothesis testing, where each junction is tested multiple times across the dataset. To address this issue, SICILIAN adopts a multiple hypothesis testing correction strategy where for each junction, it collects the empirical *p* values (*emp.p*’s) across the samples from an individual and then computes its median (or the “SICILIAN score”) as a unified criterion to decide whether the junction should be called across all 10x samples or SS2 cells (Fig. 1C). We took the median because as the number of random variables increases, their median converges to their expectation; therefore, the median can be used as a consistent statistical measure that controls for multiple hypothesis tests of a junction being an artifact across samples. With this approach, if a junction possesses a significant *emp.p* in one sample just by chance but there is enough evidence in other samples that the junction is a false positive, SICILIAN would be able to correct the call by considering the junction’s *emp.p*’s in other samples, which would lead to a large median and consequently the removal of the junction. Using this system, each junction will be called consistently across all samples. For this paper, we used a cutoff of 0.15 for the SICILIAN score (or the median of *emp.p*) as this value optimized the Youden's index (sensitivity *+* specificity −1) on the benchmarking datasets with known ground truth.

For each sample in which junction *i* is originally present, the *emp.p* is only considered in this step if it meets several criteria: the fraction of reads with a genomic alignment that are also mapped to this junction in the sample is < 0.1, the reads mapping to the junction in this sample have different starting points for their alignment (reads aligned to a junction have different overlaps), the average length of the longest stretch of either A’s, T’s, G’s, or C’s in the reads mapping to the junction is less than 11, and the average entropy of aligned reads to the junction is greater than 3. These filters are included because any of these individual criteria provides significant evidence on its own that the junction is a false positive. If a junction “fails” any of these criteria in one sample, the *emp.p* from that sample will not be included in the set of *emp.p* used to find the median.

### Read sequence entropy

To further identify false positives by spliced aligner, the SICILIAN model also includes the entropy [30] for the aligned reads, a quantitative measure of how repetitive a sequence is. For example, the entropy of sequence TCACTCTCCCACACTCTCTCTCTCTCACACACACACACACACACACACACACACACACAC, which has many repeats of AC and TC is 2.1, while the entropy of sequence GAAAGTGTATAACTACAATCACCTAATGCCCACAAGGTACTCTGTGGATATCCCCTTGGA is 4.0. We computed the entropy for a read sequence based on the distribution of overlapping 5-mers in the sequence. For example, for ACTCCGAGTCCTCCG the list of 5-mers would be: ACTCC, CTCCG, TCCGA, CCGAG, CGAGT, GAGTC, AGTCC, GTCCT, TCCTC, CCTCC, and CTCCG. Let *k*_1_,...,*k*_*n*_ be the set of unique 5-mers in the read sequence and for any *k*_*i*_, let *N*(*k*_*i*_) be the number of times that kmer *k*_*i*_ appears in the read sequence (for example, CTCCG appears twice in ACTCCGAGTCCTCCG). We define the read entropy as $\sum \limits_{i=1}^n-\frac{N\left({k}_i\right)}{n}\log\ \left(\frac{N\left({k}_i\right)}{n}\right)$.

### Mouse lemur liftover analysis

We used the UCSC LiftOver tool [27] (https://genome.ucsc.edu/cgi-bin/hgLiftOver) to convert the coordinates of the junctions detected by SICILIAN in MLCA from mouse lemur (Mmur3) to human (hg38) genome assemblies and analyzed the annotation status of the orthologous junctions in the human transcriptome. We used the recommended settings for LiftOver and analyzed only those junctions that have been successfully and uniquely converted by the LiftOver tool.

### Cloud-based implementation for SICILIAN

Given that the SICILIAN algorithm uses a number of tools (e.g., STAR aligner and UMI-tools [31]) and Python and R packages, to alleviate the installation difficulties and guarantee the reusability of our method, a reproducible workflow based on Common Workflow Language (CWL) and docker containers has been created and can be accessed on the Cancer Genomics Cloud [32]: https://cgc.sbgenomics.com/public/apps#jordanski.milos/deepest-fusion/sicilian/ (Additional file 1: Fig. S8). In addition to an immediate availability, the reproducible and ready-to-deploy implementation of SICILIAN on a cloud computational environment provides the opportunity to leverage the enormous computational power in cloud environments to achieve scalability, one of the main roadblocks in big data studies.

### File downloads

- Human RefSeq hg38 annotation file was downloaded from: https://ftp.ncbi.nlm.nih.gov/genomes/all/GCF/000/001/405/GCF_000001405.39_GRCh38.p13/GCF_000001405.39_GRCh38.p13_genomic.gtf.gz
- Mouse Lemur RefSeq Micmur3 assembly files were downloaded from: https://www.ncbi.nlm.nih.gov/assembly/GCF_000165445.2/
- The list of GTEx splice junctions was downloaded from GTEx Portal: https://storage.googleapis.com/gtex_analysis_v8/rna_seq_data/GTEx_Analysis_2017-06-05_v8_STARv2.5.3a_junctions.gct.gz

### Availability of data and materials

The 10x benchmarking dataset [20] is available on the SRA database (GSM3618014). The matched cell lines (HCC827, H1975, A549, H838, and H2228) for the benchmarking 10x dataset were downloaded from the NCI Genomic Data Commons (GDC) Legacy Archive (https://portal.gdc.cancer.gov/legacy-archive). The simulated benchmarking datasets [8] were downloaded from ArrayExpress (accession number: E-MTAB-1728). The HISAT simulated datasets [12] were downloaded from: http://www.ccb.jhu.edu/software/hisat/downloads/hisat-suppl/reads_perfect.tar.gz and http://www.ccb.jhu.edu/software/hisat/downloads/hisat-suppl/reads_mismatch.tar.gz. The human lung scRNA-seq data used here was generated through the Human Lung Cell Atlas [11] project and is accessible through European Genome-phenome Archive (accession number: EGAS00001004344). The mouse lemur single-cell RNA-seq data used in this study was generated as part of the Tabula Microcebus consortium and the fastq files were downloaded from: https://tabula-microcebus.ds.czbiohub.org. SICILIAN code is publicly available under the GNU GPL-2.0 License and can be accessed via a GitHub repository: https://github.com/salzmanlab/SICILIAN (DOI: 10.5281/zenodo.5081832) [33]. All code used for benchmarking can be found at https://github.com/salzmanlab/SICILIAN/tree/master/benchmarking. Also, a cloud-based implementation of SICILIAN with a web-based user interface based on docker containers is publicly available through the NCI-funded Cancer Genomics Cloud platform: https://cgc.sbgenomics.com/public/apps#jordanski.milos/deepest-fusion/sicilian/. All data was run on the SICILIAN version corresponding to the commit on July 9, 2020, to the SICILIAN GitHub repository with the exception of Smart-seq2 data which includes a paired-end module introduced by the commit in October 2020.

## Supplementary Information

**Additional file 1: Supplemental figures S1-S8.**

**Additional file 2.** Review history.

## References

[1] Baralle FE, Giudice J. Alternative splicing as a regulator of development and tissue identity. *Nat. Rev. Mol. Cell Biol*. 2017;18(7):437–51. 10.1038/nrm.2017.27. 10.1038/nrm.2017.27 PMC6839889 28488700

[2] Scotti MM, Swanson MS (2016). RNA mis-splicing in disease. Nat. Rev. Genet..

[3] Westoby J, Artemov P, Hemberg M, Ferguson-Smith A (2020). Obstacles to detecting isoforms using full-length scRNA-seq data. Genome Biol..

[4] Szabo L, Salzman J (2016). Detecting circular RNAs: bioinformatic and experimental challenges. Nat. Rev. Genet..

[5] Szabo L, Morey R. Statistically based splicing detection reveals neural enrichment and tissue-specific induction of circular RNA during human fetal development. *Genome Biol.* 2015;16(1):126. 10.1186/s13059-015-0690-5. 10.1186/s13059-015-0690-5 PMC4506483 26076956

[6] Dehghannasiri R, Freeman DE. Improved detection of gene fusions by applying statistical methods reveals oncogenic RNA cancer drivers. *Proc. Natl. Acad. Sci. U. S. A.* 2019;116(31):15524–33. 10.1073/pnas.1900391116 10.1073/pnas.1900391116 PMC6681709 31308241

[7] Hsieh G, et al. Statistical algorithms improve accuracy of gene fusion detection. *Nucleic Acids Res*. 2017;45(13):e126. 10.1093/nar/gkx453 10.1093/nar/gkx453 PMC5737606 28541529

[8] Engström PG (2013). Systematic evaluation of spliced alignment programs for RNA-seq data. Nat. Methods.

[9] Lebrigand K, Magnone V, Barbry P, Waldmann R (2020). High throughput error corrected Nanopore single cell transcriptome sequencing. Nat. Commun..

[10] Manipur I, Granata I, Guarracino MR (2019). Exploiting single-cell RNA sequencing data to link alternative splicing and cancer heterogeneity: A computational approach. Int. J. Biochem. Cell Biol..

[11] Travaglini KJ, Nabhan AN (2020). A molecular cell atlas of the human lung from single-cell RNA sequencing. Nature.

[12] Kim D, Langmead B, Salzberg SL (2015). HISAT: a fast spliced aligner with low memory requirements. Nature Methods.

[13] Olivieri JE, et al. RNA splicing programs define tissue compartments and cell types at single cell resolution. bioRxiv. 2021. 10.1101/2021.05.01.442281. 10.7554/eLife.70692 PMC8563012 34515025

[14] Zheng GXY, et al. Massively parallel digital transcriptional profiling of single cells. *Nat. Commun.* 2017;8(1):14049. 10.1038/ncomms14049. 10.1038/ncomms14049 PMC5241818 28091601

[15] Benegas G, Fischer J, Song YS. Robust and annotation-free analysis of isoform variation using short-read scRNA-seq data. bioRxiv. 2021. 10.1101/2021.04.27.441683.

[16] Dobin A, Davis CA (2013). STAR: ultrafast universal RNA-seq aligner. Bioinformatics.

[17] Friedman J, Hastie T, Tibshirani R (2010). Regularization Paths for Generalized Linear Models via Coordinate Descent. J. Stat. Softw..

[18] Kedzierska KZ, Gerber L (2018). SONiCS: PCR stutter noise correction in genome-scale microsatellites. Bioinformatics.

[19] Gorzynski JE, et al. High-throughput SARS-CoV-2 and host genome sequencing from single nasopharyngeal swabs. medRxiv. 2020. 10.1101/2020.07.27.20163147.

[20] Tian L, Dong X (2019). Benchmarking single cell RNA-sequencing analysis pipelines using mixture control experiments. Nat. Methods.

[21] Liu W, Zhang X (2020). Single-cell alternative splicing analysis reveals dominance of single transcript variant. Genomics.

[22] Kahles A, et al. Comprehensive Analysis of Alternative Splicing Across Tumors from 8,705 Patients. *Cancer Cell. *2018;34(2):211–224.e6. 10.1016/j.ccell.2018.07.001. 10.1016/j.ccell.2018.07.001 PMC9844097 30078747

[23] David JK, Maden SK, Weeder BR, Thompson RF, Nellore A. Putatively cancer-specific exon-exon junctions are shared across patients and present in developmental and other non-cancer cells. NAR Cancer. 2020;2(1):zcaa001. 10.1093/narcan/zcaa001. 10.1093/narcan/zcaa001 PMC8209686 34316681

[24] Tabula Microcebus Consortium. https://tabula-microcebus.ds.czbiohub.org/.

[25] Pertea M, et al. CHESS: a new human gene catalog curated from thousands of large-scale RNA sequencing experiments reveals extensive transcriptional noise. Genome Biology. 2018;19(1):208. 10.1186/s13059-018-1590-2. 10.1186/s13059-018-1590-2 PMC6260756 30486838

[26] Carithers LJ, Moore HM (2015). The Genotype-Tissue Expression (GTEx) Project. Biopreservation and Biobanking.

[27] Kuhn RM, Haussler D, Kent WJ (2013). The UCSC genome browser and associated tools. Brief. Bioinform..

[28] Larsen PA, Harris RA (2017). Hybrid de novo genome assembly and centromere characterization of the gray mouse lemur (Microcebus murinus). BMC Biol..

[29] Olivieri JE, Dehghannasiri R, Salzman J. The SpliZ generalizes ‘Percent Spliced In’ to reveal regulated splicing at single-cell resolution. *bioRxiv *2021*.*10.1101/2020.11.10.377572. 10.1038/s41592-022-01400-x PMC9089759 35241832

[30] Román-Roldán R, Bernaola-Galván P, Oliver J (1996). Application of information theory to DNA sequence analysis: A review. Pattern Recognit..

[31] Smith T, Heger A, Sudbery I. UMI-tools: Modelling sequencing errors in Unique Molecular Identifiers to improve quantification accuracy. *Genome Res. *2017;27(3):491–9. 10.1101/051755. 10.1101/gr.209601.116 PMC5340976 28100584

[32] Lau JW, et al. The Cancer Genomics Cloud: Collaborative, Reproducible, and Democratized-A New Paradigm in Large-Scale Computational Research. *Cancer Res. *2017;77(21):e3–6. 10.1158/0008-5472.can-17-0387. 10.1158/0008-5472.CAN-17-0387 PMC5832960 29092927

[33] Dehghannasiri R, Olivieri J, Salzman J. SICILIAN. GitHub. 2021. 10.5281/zenodo.5081832

