# Supplementary material for: Specific splice junction detection in single cells with SICILIAN
Source: Genome Biol. 2021 Aug 5;22:219. doi: 10.1186/s13059-021-02434-8 (PMC8339681; doi:10.1186/s13059-021-02434-8)
Supplement: Supplementary file 1 — Additional file 1: Supplemental figures S1-S8. [file 13059_2021_2434_MOESM1_ESM.pdf]

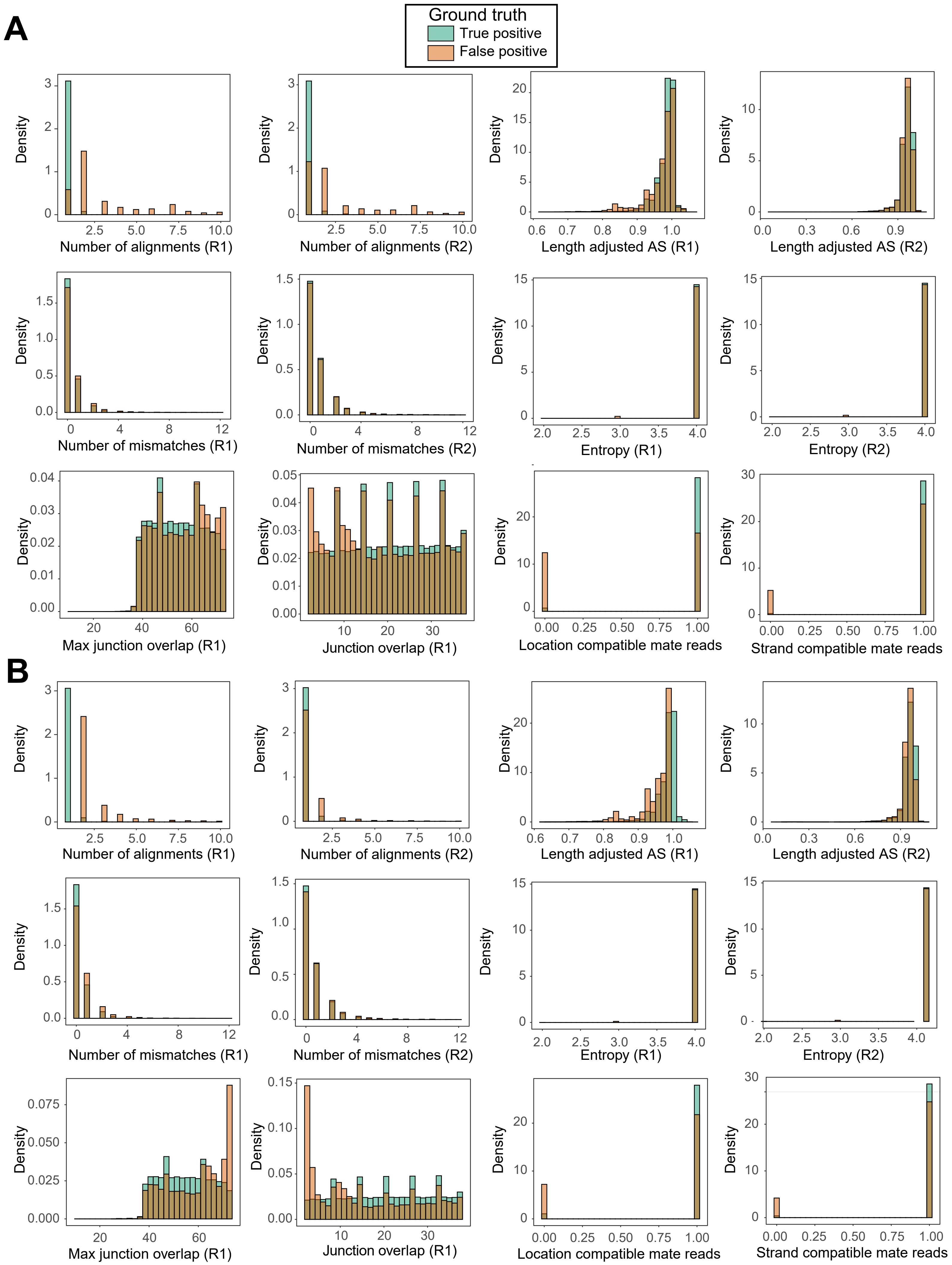

Fig S1. The power of the predictors employed in the model for distinguishing false-positive and true-positive junctions and the success of the training reads in modeling the alignment profiles of the true-/false-positive spliced alignments based on the simulated benchmarking dataset[8]. Since the dataset is a paired-end data we used alignment features from both R1 and R2 in our model. Negative reads tend to have more mismatches, lower alignment scores, shorter overlaps, more than one reported alignment, and be less likely to be strand and location compatible, which is consistent with the mapping profile of false positive spliced alignments. (A) Histograms show the distribution of the alignment features for the reads stratified by being aligned to a false-positive or true-positive junction according to the ground truth of spliced alignment for the dataset. (B) Histograms show the distribution of the alignment features used as predictors in the model for the reads stratified by being used as the positive or negative set for training the model.

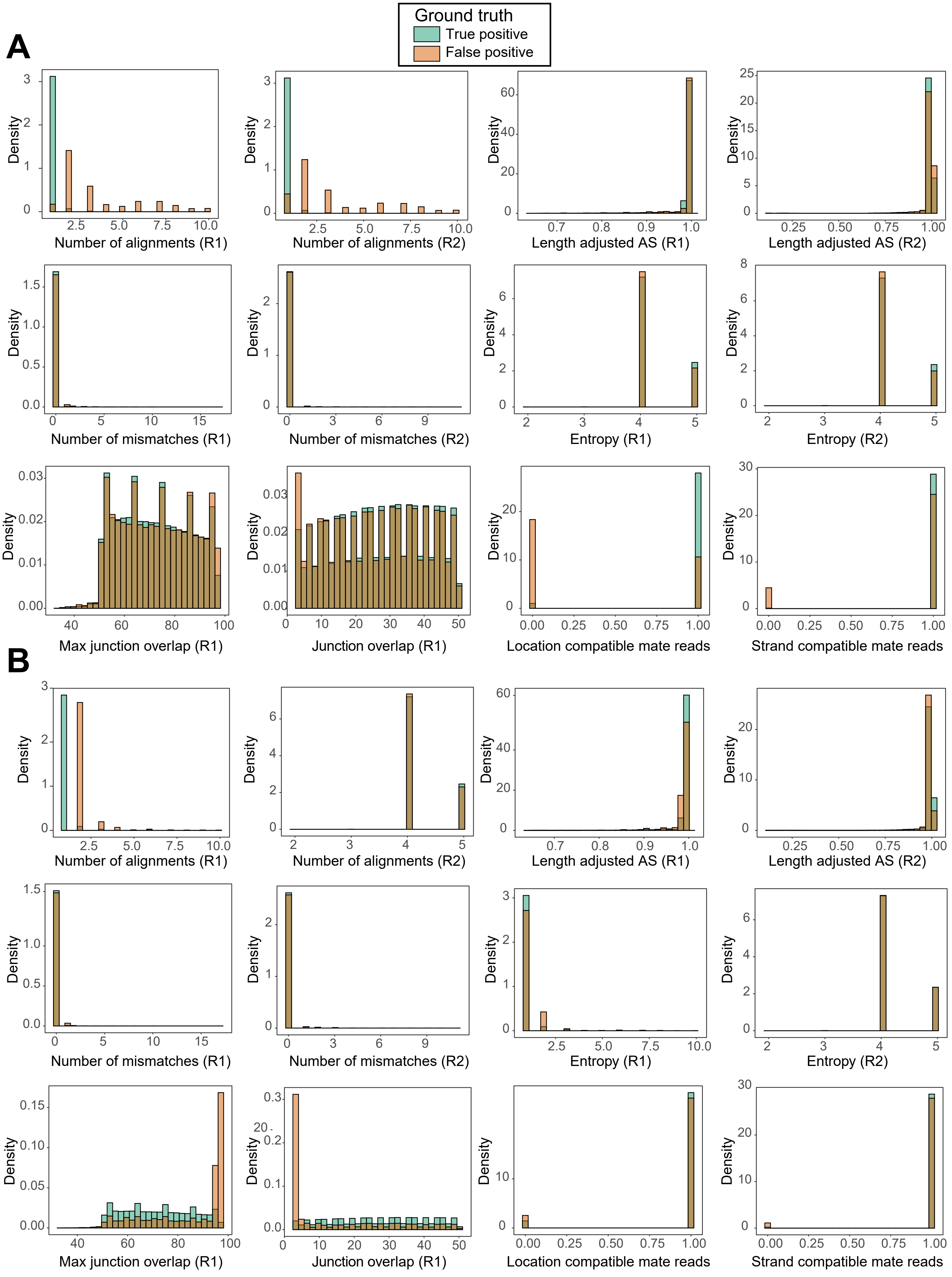

Fig S2. The same analysis as in Fig S1 based on the HISAT benchmarking dataset[12]. (A) Alignment features are stratified according to the spliced alignment being a true positive or false positive according to the ground truth. (B) Alignment features are stratified according to the spliced alignment being used in the positive or negative training set.

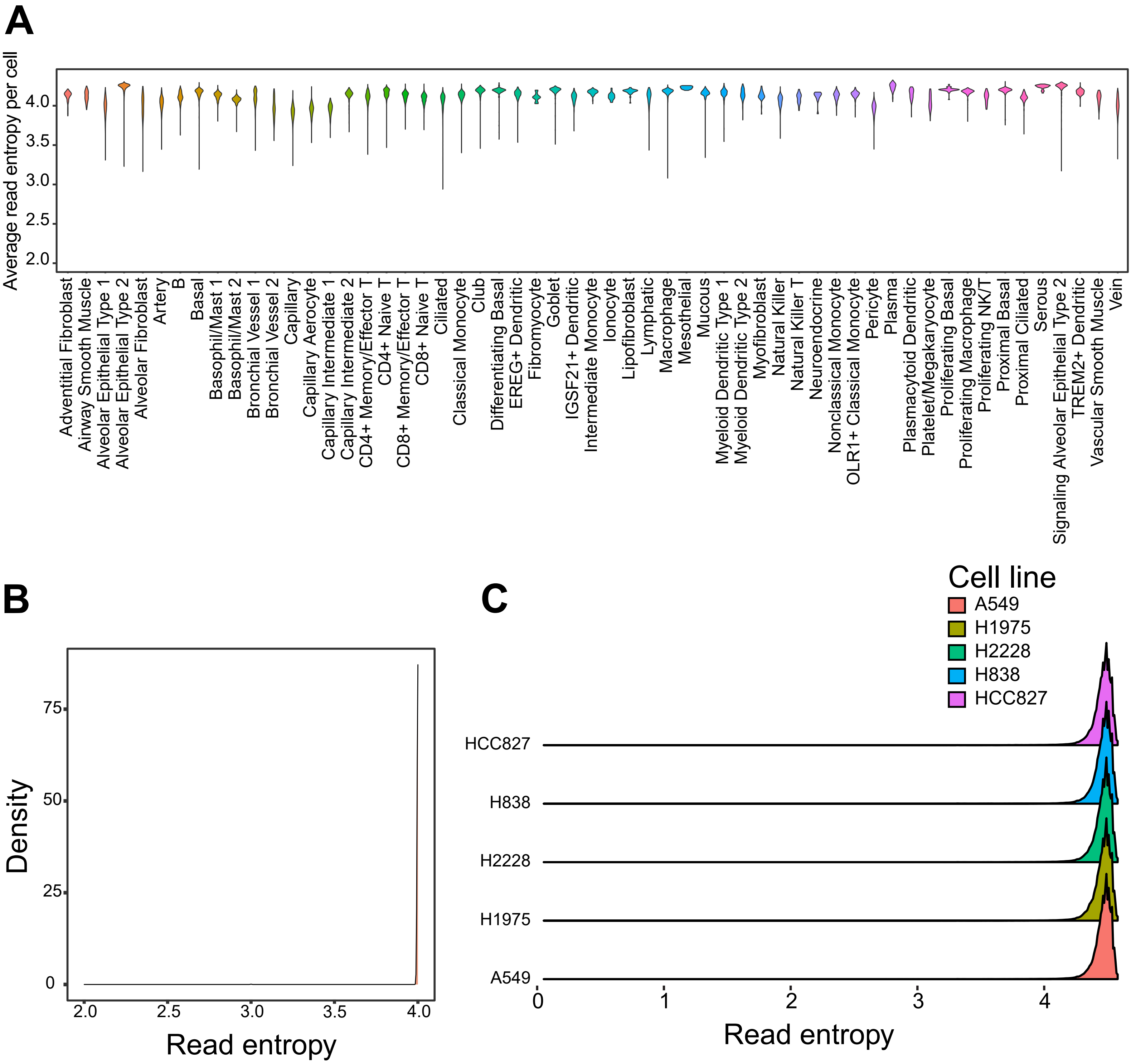

Fig S3. High variation of read sequence entropy in scRNA-Seq. (A) Each violin plot shows the average sequence entropy of the reads in each cell within a cell type in the HLCA 10x dataset. (B) Bulk simulated datasets do not model entropy variation in real data. The density plot shows the read sequence entropy in a simulated dataset[8]. (C) Low entropy reads in real bulk RNA-seq datasets are less prevalent than scRNA-Seq. The plot shows the read entropy distributions in five cell lines used for generating the benchmarking single-cell dataset[20].

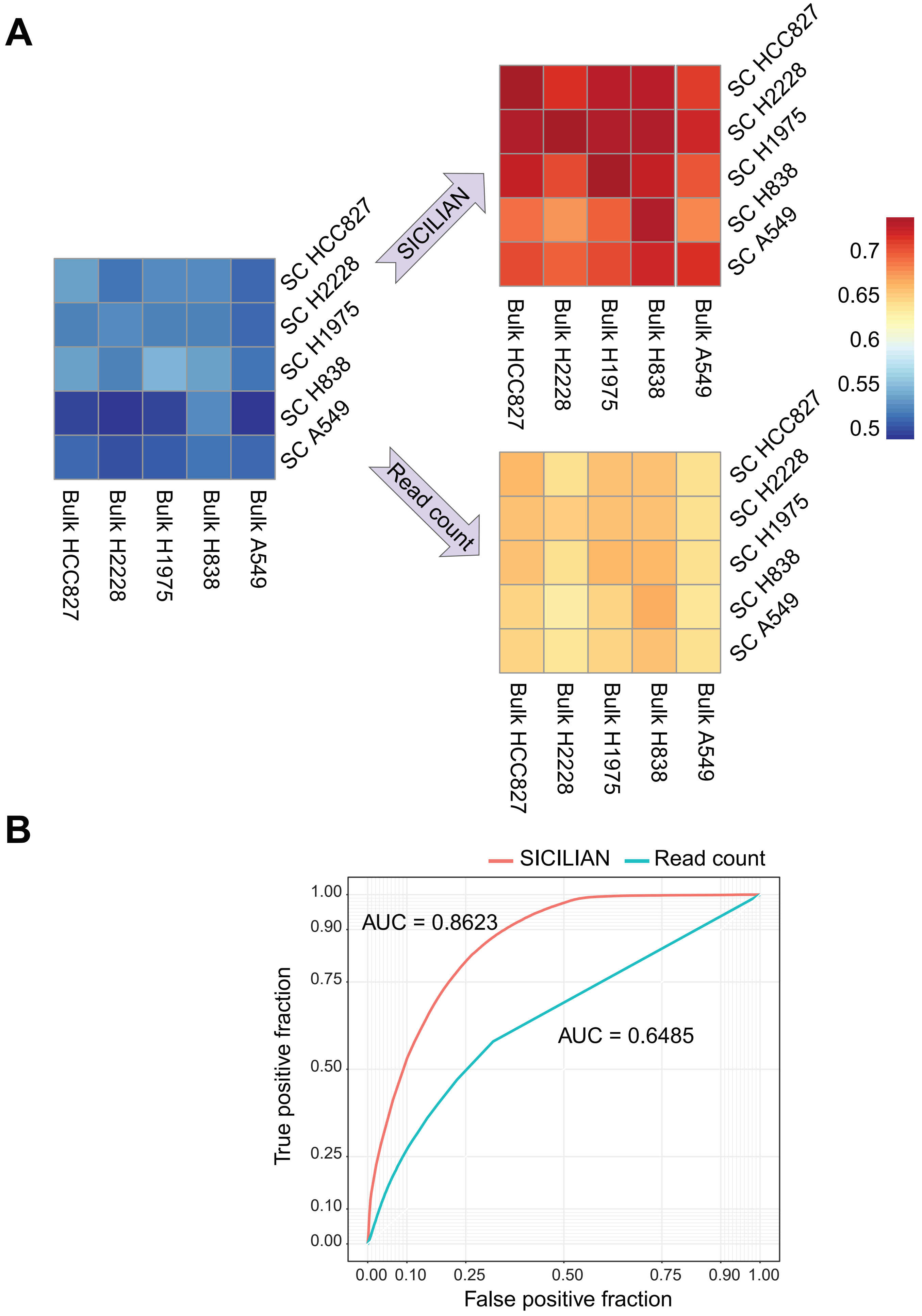

Fig S4. Benchmarking SICILIAN's performance based on the matched scRNA-seq and bulk data from five human lung adenocarcinoma cell lines[20]. (A) The heatmaps showing the fraction of called junctions in 10x cells[20] that have been found in their matched bulk cell lines. The same scale has been used for all three heatmaps. (B) The ROC curves by SICILIAN and read-count-based criterion for the matched single-cell and bulk datasets[20] show that SICILIAN achieves a higher AUC compared to that of the read-count-based criterion. For generating these ROC curves, we considered a junction for a single cell as a true positive call if it is also called in the corresponding bulk cell line of that single cell and otherwise would be counted as a false positive.

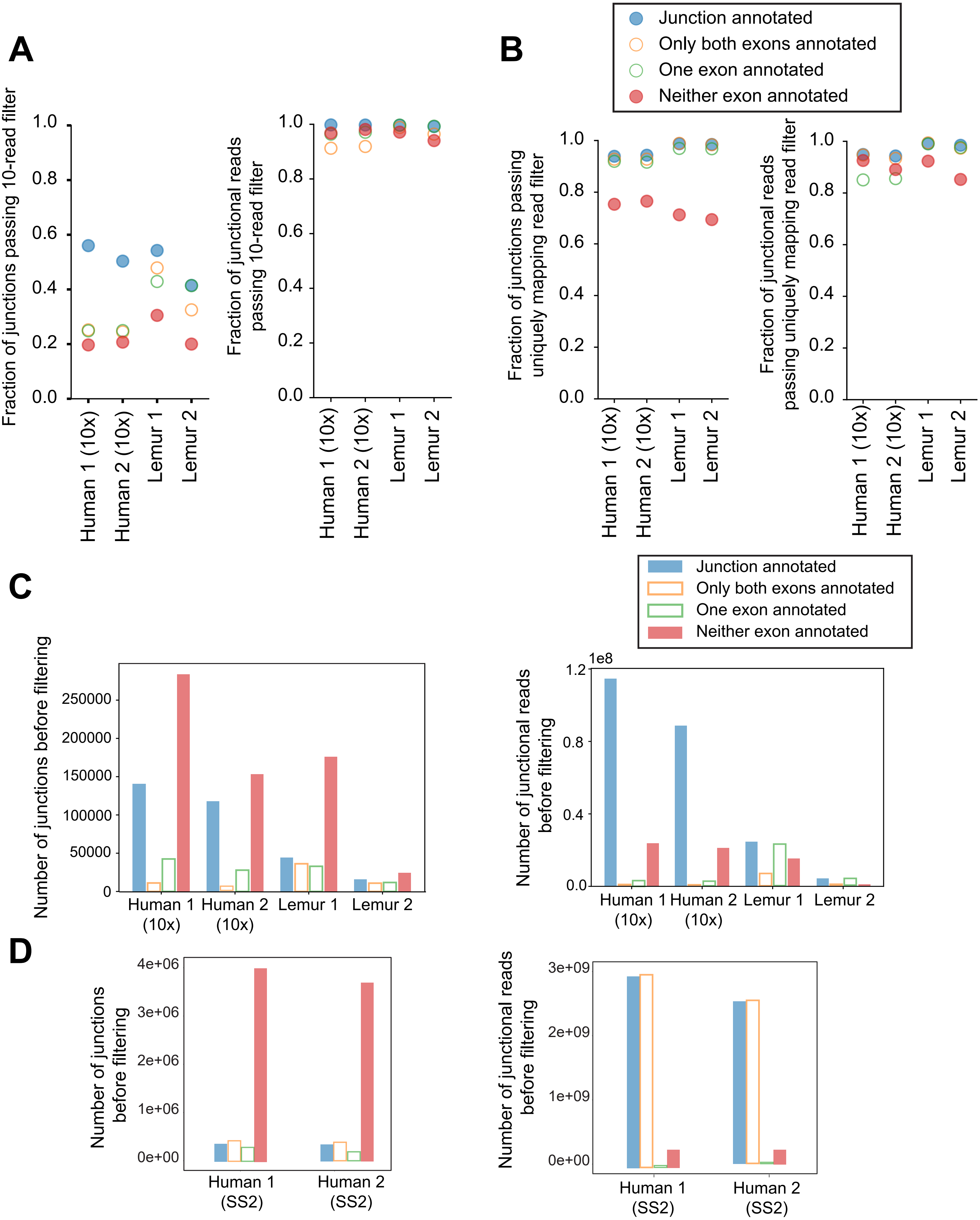

Fig S5. Annotation status of the junctions and junctional reads called by SICILIAN and the read-count criterion for HLCA 10x and SS2 datasets. (A) Using a 10-read-count threshold causes fewer annotated junctions to be called, and many more unannotated junctional reads to be called (only junctions with at least two reads in the given dataset are plotted). (B) Including all junctions that have at least one read uniquely mapping to them causes a high fraction of unannotated junctions and unannotated junctional reads to be called (only junctions with at least two reads in the given dataset are plotted). (C) The number of unannotated junctions is greater than the number of annotated junctions before filtering in all individuals. Lemur individuals have a higher proportion of junctions with one or both exons annotated but without the junction annotated. (D) The same plots as in (C) but for HLCA SS2 datasets.

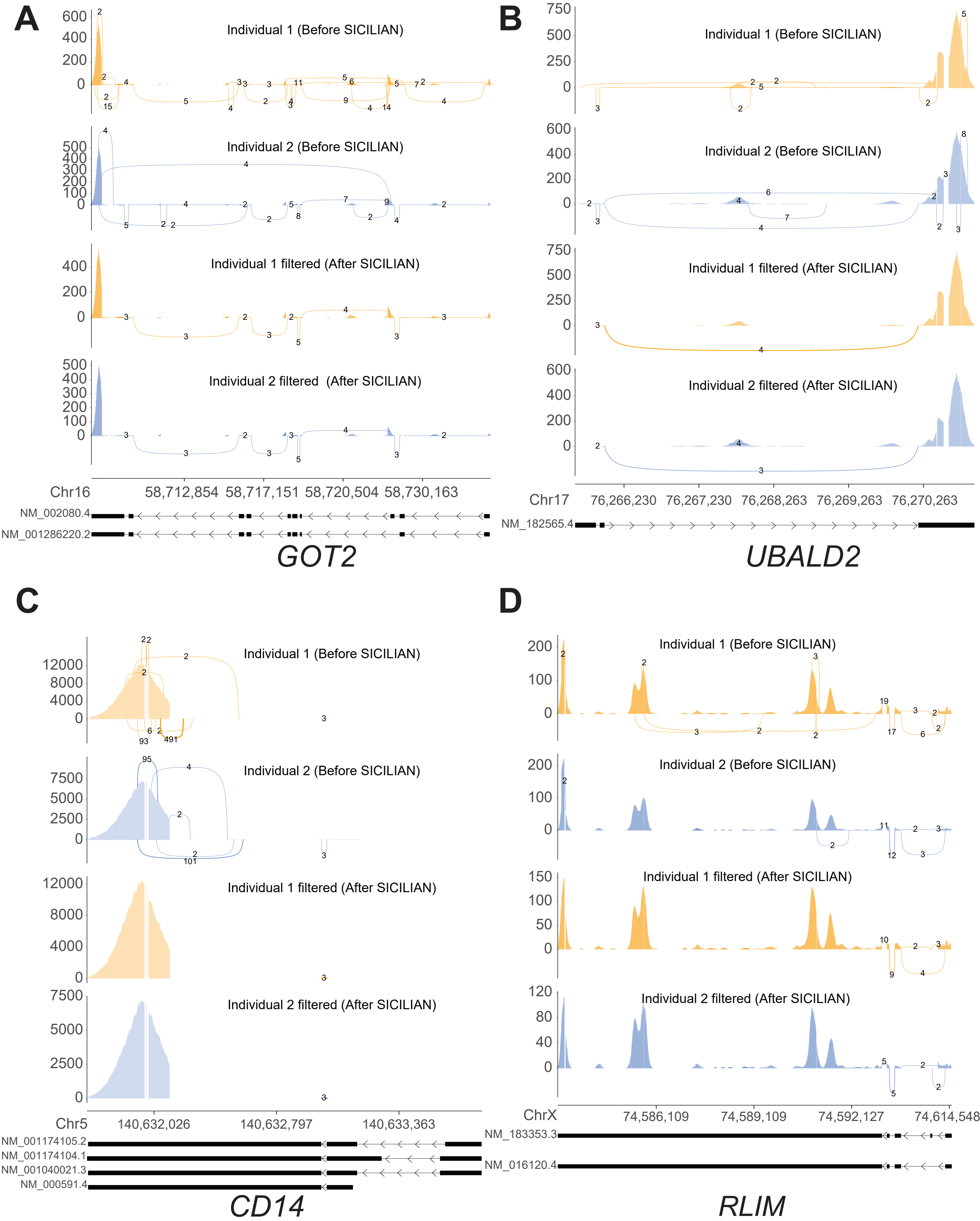

Fig S6. The ability of SICILIAN to remove noisy false-positive junctions as shown by the sashimi plots for some genes. Sashimi plots for genes (A) *GOT2*, (B) *UBALD2*, (C) *CD14*, and (D) *RLIM* suggest that SICILIAN was able to identify noisy unannotated junctions while keeping all annotated junctions in an unbiased manner. For each gene, there are two sashimi plots before applying SICILIAN (for the two individuals in the HLCA 10x dataset) and two sashimi plots after applying SICILIAN. Sashimi plots show the aggregated splice junctions and read coverage across all 10x samples for each individual.

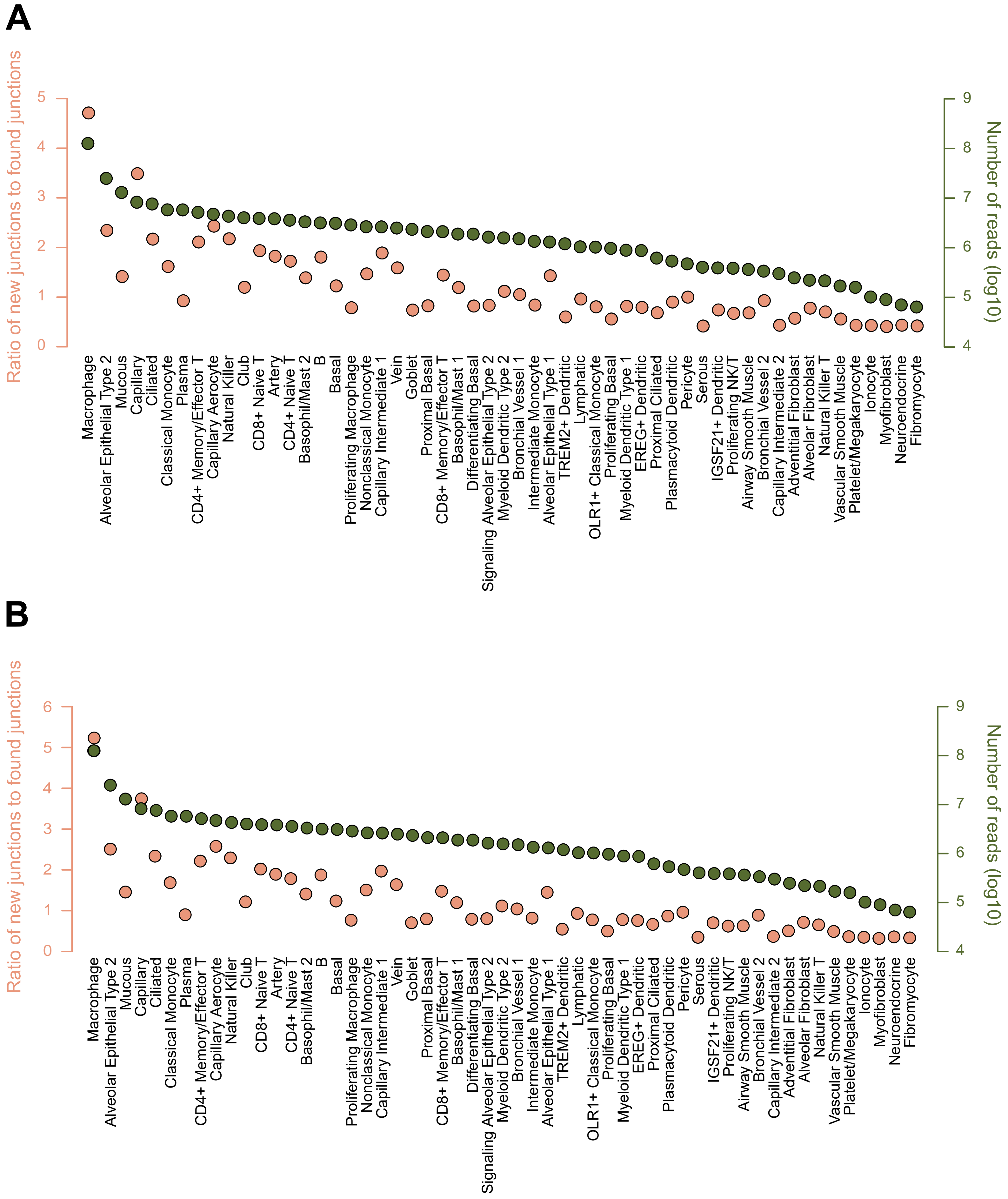

Fig S7. Comparison of the junctions found in the HLCA dataset with two human splicing databases: (A) CHESS[25] and (B) GTEx[26]. For each cell type within the HLCA data, the plot shows the ratio of the number of junctions that are not found in the database (defined as new junctions) to the number of junctions that are found in the database (defined as found junctions). The green dots show the number of junctional sequencing reads (in the logarithmic scale) for each cell type.

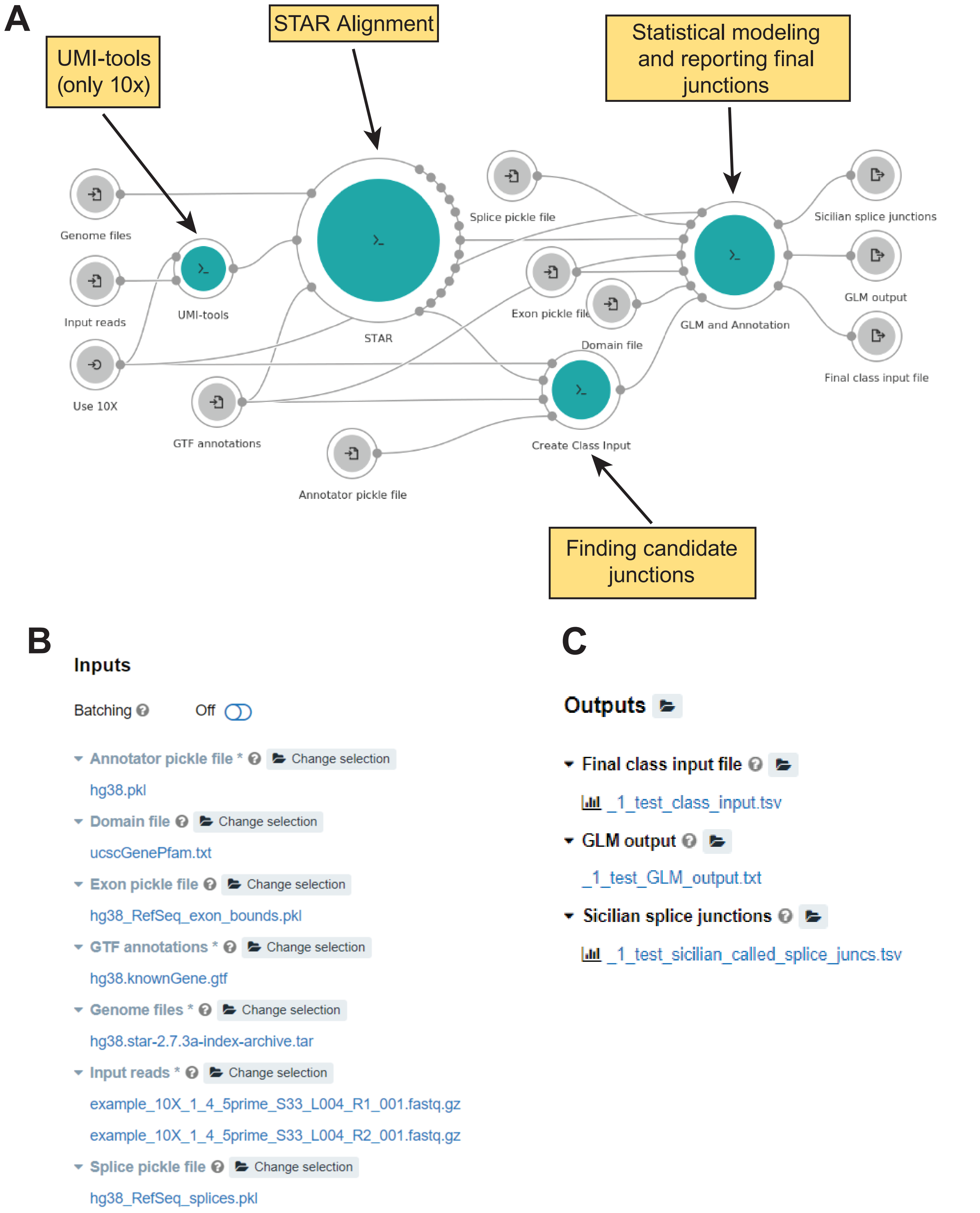

Fig S8. Dockerized cloud-based implementation of SICILIAN on the Cancer Genomics Cloud platform. (A) The underlying workflow of the implemented app, which consists of four main apps: the UMI-tools[31] app (executed only when the input data is 10x), the STAR[16] app for conducting spliced alignment, the Create class input app for extracting candidate junctions and their alignment information from the STAR BAM file, and the GLM and Annotation app for the statistical modeling and reporting junctions. (B) Input files needed for running the app (i.e., RNA-Seq data and needed index and reference files). (C) Output files after running the app: Class input file (spliced alignment information), GLM output file (all junctions with their statistical scores), and SICILIAN splice junctions (final called junctions with their exon, splice, and protein domain annotations).
