# Supplementary material for: Specific splice junction detection in single cells with SICILIAN
Source: Genome Biol. 2021 Aug 5;22:219. doi: 10.1186/s13059-021-02434-8 (PMC8339681; doi:10.1186/s13059-021-02434-8)
Supplement: Supplementary file 2 — Additional file 2. Review history. [file 13059_2021_2434_MOESM2_ESM.docx]

Review History

**First round of review**

**Reviewer 1**

**Are you able to assess all statistics in the manuscript, including the appropriateness of statistical tests used?**

Yes, and I have assessed the statistics in my report.

**Comments to author:**

In this report, Dehghannasiri and colleagues describe a tool for analysis of splicing from single-cell RNA-Seq performed with 10x technology. This is a potentially valuable contribution to enable the transcriptomics field to make novel discoveries about splicing with these new technologies.

The authors propose a procedure for identifying false positive junction calls from 10x data. This is necessary because the starting set of junction calls has a high error rate (or high noise), with <10% of them shared with annotation (Fig 2F). The authors miss an opportunity to describe why this initial error rate is so high, and what is the possibility that the technology may detect rare or unusual bona-fide splicing. For example, are all chimeric alignments and non GT-AG adjacent gapped alignments preserved for the initial set? If these are a-priori low likelihood to represent real events does it make sense to remove them at the outset before SICILIAN, or does the 10x technology afford the possibility to detect rare or aberrant splicing?

A more granular analysis of false-positives and what characterize them would be a good addition. Is the full model necessary to identify them all, or is one criterion sufficient to perform well? Also, can changing alignment parameters mitigate a lot of the problems rather than post-hoc filtering?

Specific comments for the figures:

Figure 1B. It is not clear from this panel whether a classical multiple hypothesis testing correction (eg Benjamini-Hochberg) is being applied, or if taking the median of empirical p-values, as described in the methods.

Figure 1C is cited out of order in the introduction, and it's not clear what it is showing. what is the numerator/denominator of the fraction?

Figure 1D is meant to show an improvement in concordance between two filtering methods, but the heatmaps presented do not illustrate this, since they are given in three different scales for the same colors. A single scale should be used if a difference is to be illustrated.

In Figure 1E, The ROC curves based on ground truth are based on simulations for bulk data and do not reflect the performance of the tool for its intended data/purpose. If simulated 10x data are not available, I understand this is difficult to generate.

Figure 2A serves to show that unannotated junctions are more likely filtered out, which is not surprising, and would likely also be the case with the read count criterion.

Figure 2B is intended to show the results of filtering,and show a putative novel junction. There are several problems with this panel. First, it may be showing the gene model for NTN4 (on the opposite strand), rather than PGAM1P5 as stated in the legend. The panel doesn't clearly show if it is the 5' or 3' end of the transcript. The splice junctions cannot be lined up the exon structure depicted to know if they correspond to exon boundaries. There is no directionality or strandedness given which would aid determination whether they derive from this transcript. A coverage track to see exon coverage is also necessary. This panel is important because the paper lacks real examples of pass/fail read alignments, which is critical to evaluate the method.

In Figure 2F, it seems to show that after SICILIAN, more than half of the remaining detected junctions are absent from annotation. If this is in fact the case, can you comment on whether annotation is really that incomplete, or is splicing more biologically noisy than appreciated?

The panels presented in Fig 2 should be re-ordered (D,E before F,G)

General comments:

Several citations are made to preprints where peer-reviewed versions are available

**Reviewer 2**

**Are you able to assess all statistics in the manuscript, including the appropriateness of statistical tests used?**

Yes, and I have assessed the statistics in my report.

**Comments to author:**

Dr. Salzman and colleagues report SICILIAN, a new computational tool for improving splice junction detection in single-cell RNA-seq (scRNA-seq) data. The idea of using multiple features of spliced alignment & read mapping to improve splice junction detection is interesting, and the results appear promising. However, the current manuscript has numerous writing and technical issues that need to be addressed in a substantially revised version.

Major issues:

1. A major issue is that the manuscript is not written in a conceptually intuitive and accessible manner. The Introduction section of the manuscript is too short, and the motivation for the proposed method is not adequately justified. Splice junction detection from short-read RNA-seq data is an old problem. The authors justified the development of SICILIAN based on the argument that scRNA-seq data are more prone to artifacts and single-cell-specific noise. However, this is described in a vague manner, and there is no clear explanation for what exactly these artifacts are, and how they are related to specific features of scRNA-seq experiments. I think this part of the introduction needs to be substantially expanded, so readers can appreciate the motivation for this work before getting into technical details. It is important to tell the readers what exactly these scRNA-seq artifacts are and how they potentially influence the detection of splice junctions.

2. A second major issue is the apparently quite arbitrary definition of gold-standards/true positives/false positives. For example, to train the penalized generalized linear model, the authors defined positive and negative training classes based on whether each junctional read also has a genomic alignment. This seems like a quite inaccurate way of defining positive and negative training labels. The lack of a genomic alignment for a putative junctional read does not mean it's a bona fide junctional read. Similarly, when evaluating SICILIAN using matched single-cell and bulk datasets, the authors defined junctions detected in the single-cell and bulk datasets of the same cell line as true positives. If I understand this correctly, this is basically using a subset of events detected by a given tool as the gold-standard to evaluate that tool. Finally, in the analysis of the lung data, the authors used annotated and unannotated junctions as surrogates for true positive and false positive junctions. This basically means any bona fide novel splice junction detected from the data will be automatically classified as a false positive. Overall, the lack of consistency and rigor in defining gold-standards is a recurring issue in the manuscript, that needs to be addressed.

3. Read entropy seems like an important feature for the SICILIAN model, but there is no intuitive explanation for what it is in the Results section when the term is first introduced. There is a detailed explanation in Methods, but I think that comes too late.

4. A major novelty of SICILIAN is to statistically evaluate the confidence of the alignment for each junctional read, using multiple features of spliced alignment & read mapping. It would be very useful to assess which specific features make major contributions to the improved splice junction detection as reported for SICILIAN. The authors should consider dropping one feature at a time, re-train the model, and evaluate feature importance based on the drop in performance.

5. I do not understand the rationale for the multiple hypothesis testing correction in SICILIAN. The authors seem to think that splice junctions detected across multiple scRNA-seq samples are more reliable. While this certainly makes sense, implementing this as a filtering step means that condition-specific or individual-specific splice junctions would be filtered out, which does not seem like a good idea.

6. SICILIAN was developed and tested entirely based on 10X datasets. To show the general applicability of this tool for splice junction detection from scRNA-seq data, the authors should demonstrate its use on data generated by other short-read protocols, such as Smart-seq2/3.

7. In Figure 1E, the improved performance of SICILIAN on the bulk-level simulated datasets seems too large to be true. The authors have already stated that SICILIAN is designed to address artifacts specific to scRNA-seq experiments, so why is there such a large increase in AUC value on bulk-level simulated datasets? Is there something wrong with the datasets or the evaluation? This also circles back to a key conceptual question about this work - what is the uniqueness of SICILIAN for scRNA-seq data as compared to bulk-level RNA-seq data?

Minor issues:

1. Page 2, Figure 1C appears out of order in the main text, before Figure 1A and 1B are referenced. This needs to be fixed.

2. Figure 1A, the diagram for "Junction-level statistical evidence collection" looks odd. None of the spliced alignments shown in the diagram appear to support the splice junction.

3. Page 4, first paragraph: why do these numbers not add up to 100%? The description of results in this paragraph is somewhat confusing.

4. Page 5, first paragraph: the authors discussed the potential application of SICILIAN to long-read RNA-seq data. This seems like a stretch and should be taken out, unless supported by data. The features and error profiles of long reads are very different from short reads.

**Authors Response**

**Point-by-point responses to the reviewers’ comments:**

Reviewer reports:

Reviewer #1: In this report, Dehghannasiri and colleagues describe a tool for analysis of splicing from single-cell RNA-Seq performed with 10x technology. This is a potentially valuable contribution to enable the transcriptomics field to make novel discoveries about splicing with these new technologies.

*-	We appreciate your feedback that our proposed method is a highly valuable contribution to the field of scRNA-Seq.*

The authors propose a procedure for identifying false positive junction calls from 10x data. This is necessary because the starting set of junction calls has a high error rate (or high noise), with <10% of them shared with annotation (Fig 2F). The authors miss an opportunity to describe why this initial error rate is so high, and what is the possibility that the technology may detect rare or unusual bona-fide splicing.

*-	We appreciate your comment. The error rate is so high because aligners are only computational methods and do not consider biochemical or sequencing artifacts as has been shown by other benchmarking studies(Engström et al. 2013) and our own benchmarking analysis in this paper. Our model is totally unbiased and never uses annotation information for calling a junction, therefore if a truly expressed novel splice junction is sequenced, our model has the potential to call it. In fact, we (and others in other works) have used SICLIAN to detect substantial numbers of un-annotated junctions in 10x data. To address your well-taken point, we added the following explanation to the draft on why spliced aligners suffer from high false positive rates (Page 1, last paragraph):*

*“	Current spliced aligners call many false-positive spliced junctions, partly because they are computational procedures operating on noisy observations of expressed RNA. The factors influencing this noise include sequence properties of the genome: repetitive genomic sequence within and between genes, biochemical noise introduced during library preparation which could cause mismatches, template switches, and technical noise causing base call errors during sequencing. Differences between the reference and sequenced transcriptomes caused by polymorphisms and other genetic variations yield further false positives during the process of alignment. False positive alignments are further exacerbated in scRNA-seq due to higher levels of biochemical noise specific to single-cell preparations (i.e., higher prevalence of low-entropy-reads in scRNA-Seq as discussed below) as well as statistical issues: false positive splice junction calls are more prevalent in larger datasets. Together, these challenges combine to generate an ongoing debate regarding whether 10x Chromium (10x) can be used for reliable de novo splice junction detection, despite the presence of a large number of junctional reads in the datasets generated by this protocol (Figure 1A).*

*The resolution and massive number of available scRNA-seq datasets hold great promise for discovering regulatory and functional splicing biology, including (a) identifying novel un-annotated regulated alternative splicing in rare cell types present even in well-annotated organisms such as human and (b) de novo prediction of dysregulated splicing in single cell data from diseases such as in cancer or neurodegenerative diseases; (c) automatic high-quality junction prediction from poorly annotated organisms such as new model organisms.”*

For example, are all chimeric alignments and non GT-AG adjacent gapped alignments preserved for the initial set? If these are a-priori low likelihood to represent real events does it make sense to remove them at the outset before SICILIAN, or does the 10x technology afford the possibility to detect rare or aberrant splicing?

*-	Thank you for this question about the method. We have not been focused on chimeric alignments, and therefore our model does not attempt to detect them. In an ongoing work, we are expanding SICILIAN modeling for detecting chimeric junctions (such as circRNAs and fusion junctions) in scRNA-Seq. We added this to the last paragraph of the draft to emphasize that the current draft is only focused on linear splice junctions: “Additionally, we anticipate that this framework can be expanded to detect nonlinear RNA variants such as gene fusions and circRNAs using chimeric alignments reported by the aligner.” SICILIAN’s modeling is agnostic to the junction motif and takes a unified approach towards both canonical GT-AG and non-canonical junctions. We believe that removing all non-canonical junctions without taking an unbiased systematic approach would create biases and reduce sensitivity specifically given that there are a considerable number of true-positive non-canonical junctions in the transcriptome, including those processed by the minor spliceosome. Therefore, a non-canonical junction can be called by SICILIAN, if it is truly expressed and as a result possesses a reliable alignment profile. One of the main advantages of our model is that it takes an unbiased approach towards unannotated or non-canonical junctions. Therefore, SICILIAN is a perfect fit for the studies where unannotated junctions (when the organism is poorly annotated such as mouse lemur) or non-canonical junctions (to study the effect of minor spliceosome defect on the transcriptome) are the main focus.*

A more granular analysis of false-positives and what characterize them would be a good addition. Is the full model necessary to identify them all, or is one criterion sufficient to perform well? Also, can changing alignment parameters mitigate a lot of the problems rather than post-hoc filtering?

*-	We would like to thank the reviewer for this comment. We have now added two additional figures to the supplement (Suppl. Figures 1 and 2) which show the distributions of the various alignment parameters used as predictors in our modeling where reads are stratified either based on being positive/negative training reads or being mapped to true positive/ false positive junctions according to the ground truth, which is available for the simulated data. We also added the following to the text (Page 3, first paragraph):*

*“These predictors have power to distinguish reads aligned to false-positive and true-positive junctions defined by ground truth from simulated datasets. The positive and training reads used for training the model can reliably model the general profile of the true-positive and false-positive reads (Suppl. Figures 1,2). ”*

*Additionally, these histograms suggest that the mapping profile of the true positive and false positive reads are not completely disjoint, therefore using a univariate model based on only one of the predictors would lower the precision. For example, we showed in Suppl. Figure 5B that including all junctions with uniquely mapping reads would result in calling a large fraction of unannotated junctions (which are likely false positives).*

Specific comments for the figures:

Figure 1B. It is not clear from this panel whether a classical multiple hypothesis testing correction (eg Benjamini-Hochberg) is being applied, or if taking the median of empirical p-values, as described in the methods.

*-	We appreciate your comment (the figure has now changed to Figure 1C in the revised draft). We do not use classic correction approaches and use the median emp.p to leverage the information for each junction across all samples in the dataset. We have now clarified this in the revised figure.*

Figure 1C is cited out of order in the introduction, and it's not clear what it is showing. what is the numerator/denominator of the fraction?

*-	Thank you for pointing this out. This figure (now Figure 1A) is meant to show that a large fraction of reads in 10x scRNA-Seq data are in fact splice reads, suggesting the potential of scRNA-Seq techniques for splicing analysis, a promise that has not been achieved due to the challenges of precise detection of splice junctions in scRNA-Seq. We have now changed the axis label to be “fraction of junctional reads out of all reads per cell”.*

Figure 1D is meant to show an improvement in concordance between two filtering methods, but the heatmaps presented do not illustrate this, since they are given in three different scales for the same colors. A single scale should be used if a difference is to be illustrated.

*-	We appreciate your comment. We have added the heatmaps with the same scale to the supplement (Suppl. Figure 4A) and decided to keep the original heatmaps in Figure 1 as it better conveys the differences between the read count and SICILIAN criteria. We have depicted the scales in the figure and we had already explained the fact that there are global increases in concordance in the first submission’s text: “SICILIAN increases the concordance between the detected junctions from 10x and bulk RNA-seq regardless of the pairs’ cells of origin, which is consistent with SICILIAN identifying and removing scRNA-seq-specific artifacts.”*

In Figure 1E, The ROC curves based on ground truth are based on simulations for bulk data and do not reflect the performance of the tool for its intended data/purpose. If simulated 10x data are not available, I understand this is difficult to generate.

*-	We would like to thank the reviewer for this comment. To our knowledge, there is no simulated scRNA-Seq data with groundtruth. That is why we resorted to the simulated bulk data with known ground truth to be able to systematically benchmark our method.*

Figure 2A serves to show that unannotated junctions are more likely filtered out, which is not surprising, and would likely also be the case with the read count criterion.

*-	We agree with the reviewer that the read count criterion can also filter out unannotated junctions; however, when we directly compared the read count criterion against the SICILIAN score (Figure 2C), the SICILIAN score achieved a much higher AUC compared to that of the read count criterion (0.74 vs 0.5), suggesting the higher precision of the SICILIAN for removing likely FP unannotated junctions. We added this explanation to the second paragraph on page 4:*

*“Note that due to the power of scRNA-seq in capturing cells from rare cell types, if there are unannotated junctions with low read counts in these rare cell types, we would not be able to detect those junctions based on a read-count criterion and need a better detection regime such as SICILIAN for calling them.”*

Figure 2B is intended to show the results of filtering,and show a putative novel junction. There are several problems with this panel. First, it may be showing the gene model for NTN4 (on the opposite strand), rather than PGAM1P5 as stated in the legend. The panel doesn't clearly show if it is the 5' or 3' end of the transcript. The splice junctions cannot be lined up the exon structure depicted to know if they correspond to exon boundaries. There is no directionality or strandedness given which would aid determination whether they derive from this transcript. A coverage track to see exon coverage is also necessary. This panel is important because the paper lacks real examples of pass/fail read alignments, which is critical to evaluate the method.

*-	We would like to thank the reviewer for pointing this out. We have now fixed the figure and it now shows the splice junctions and exon coverage for gene GDI1 and based on the reviewer’s suggestion we provided more real examples by adding the sashimi plots for few other genes to the supplement (Suppl. Figure 6) and added the following to the text:*

*“For many genes, including GDI1, GOT2, and CD14, SICILIAN removed all noisy unannotated junctions and kept only annotated junctions although the algorithm was agnostic to the annotation (Figure 2B, Suppl. Figure 6)”*

In Figure 2F, it seems to show that after SICILIAN, more than half of the remaining detected junctions are absent from annotation. If this is in fact the case, can you comment on whether annotation is really that incomplete, or is splicing more biologically noisy than appreciated?

*-	We appreciate the interest in the large fraction of junctions called by SICILIAN that are unannotated. We hypothesize several potential causes for this. First, even for deeply-studied organisms such as human, transcriptome annotations are not perfect. Second, deep scRNA-Seq studies enable us to study RNA isoforms in rare cell types that might be missed from the average tissue-level RNA expression captured by bulk RNA sequencing, which is the common practice used for inferring transcriptome annotation. Therefore, scRNA-Seq is equipped to potentially capture junctions that are usually missed by bulk RNA-Seq as has been shown by Figure 2F where half of the junctions were missed from two bulk-level splice junction databases. Third, individual- or condition-specific splice junctions might not be present in the annotation. Fourth, splicing is estimated to be a noisy process: it has been estimated that 99% of the transcriptional variety produced in cells has no function(Pertea et al. 2018). Although the question of how much of the unannotated splicing we identify can be attributed to each of these factors is very interesting, a full investigation is outside the scope of this manuscript.*

The panels presented in Fig 2 should be re-ordered (D,E before F,G)

*-	Thank you for pointing out this order discrepancy in the figures. We have now switched the order of the figures so that they are referenced sequentially in the text.*

General comments:

Several citations are made to preprints where peer-reviewed versions are available.

*-	Thank you for pointing this out; we have now updated all citations to the most recent available version of the papers.*

Reviewer #2: Dr. Salzman and colleagues report SICILIAN, a new computational tool for improving splice junction detection in single-cell RNA-seq (scRNA-seq) data. The idea of using multiple features of spliced alignment & read mapping to improve splice junction detection is interesting, and the results appear promising.

*-	We appreciate the reviewer's comment on the novelty and promising performance of SICILIAN and soundness of our results.*

However, the current manuscript has numerous writing and technical issues that need to be addressed in a substantially revised version.

Major issues:

1. A major issue is that the manuscript is not written in a conceptually intuitive and accessible manner. The Introduction section of the manuscript is too short, and the motivation for the proposed method is not adequately justified. Splice junction detection from short-read RNA-seq data is an old problem. The authors justified the development of SICILIAN based on the argument that scRNA-seq data are more prone to artifacts and single-cell-specific noise. However, this is described in a vague manner, and there is no clear explanation for what exactly these artifacts are, and how they are related to specific features of scRNA-seq experiments.

*-	We would like to thank the reviewer for this important comment on clarifying the motivation for developing SICILIAN. Since we have submitted this manuscript as a short report, we have limited space for the entire text and could not dedicate more space to the introduction. However, given the space consideration, we now tried to better clarify the main motivation of the paper by adding the following to the introductions section in the revised draft (second and third paragraphs in the main text):*

*“Current spliced aligners call many false-positive spliced junctions, partly because they are computational procedures operating on noisy observations of expressed RNA. The factors influencing this noise include sequence properties of the genome: repetitive genomic sequence within and between genes, biochemical noise introduced during library preparation which could cause mismatches, template switches, and technical noise causing base call errors during sequencing. Differences between the reference and sequenced transcriptomes caused by polymorphisms and other genetic variations yield further false positives during the process of alignment. False positive alignments are further exacerbated in scRNA-seq due to higher levels of biochemical noise specific to single-cell preparations (i.e., higher prevalence of low-entropy-reads in scRNA-Seq as discussed below) as well as statistical issues: false positive splice junction calls are more prevalent in larger datasets. Together, these challenges combine to generate an ongoing debate regarding whether 10x Chromium (10x) can be used for reliable de novo splice junction detection, despite the presence of a large number of junctional reads in the datasets generated by this protocol (Figure 1A).*

The resolution and massive number of available scRNA-seq datasets hold great promise for discovering regulatory and functional splicing biology, including (a) identifying novel un-annotated regulated alternative splicing in rare cell types present even in well-annotated organisms such as human and (b) de novo prediction of dysregulated splicing in single cell data from diseases such as in cancer or neurodegenerative diseases; (c) automatic high-quality junction prediction from poorly annotated organisms such as new model organisms.”

I think this part of the introduction needs to be substantially expanded, so readers can appreciate the motivation for this work before getting into technical details.

*-	Thank you for the suggestion to substantially expand the introduction. We have now modified/added two paragraphs to the introduction section (as mentioned above) so that it can better reflect the motivation for developing SICILIAN and why having a precise method for splice junction is foundational to any downstream splicing analysis in scRNA-Seq.*

It is important to tell the readers what exactly these scRNA-seq artifacts are and how they potentially influence the detection of splice junctions.

*-	We appreciate the reviewer's comment. A large fraction of 10x and Smart-seq2 reads cannot be*

*aligned due to the unique biochemical noise in these platforms. Also as we have shown in Suppl. Fig 3, reads with low entropy are more prevalent in scRNA-Seq. Entropy is one of the predictors we have used in our model which has shown to be highly effective in identifying FP junctions. We have added this explanation to the second paragraph in the text:*

*“False-positive alignments are further exacerbated in scRNA-seq due to higher levels of biochemical noise specific to single-cell preparations (i.e., higher prevalence of low-entropy-reads in scRNA-Seq as discussed below) as well as statistical issues: false-positive splice junction calls are more prevalent in larger datasets. Together, these challenges combine to generate an ongoing debate regarding whether 10x Chromium (10x) can be used for reliable de novo splice junction detection, despite the presence of a large number of junctional reads in the datasets generated by this protocol (Figure 1A).”*

2. A second major issue is the apparently quite arbitrary definition of gold-standards/true positives/false positives. For example, to train the penalized generalized linear model, the authors defined positive and negative training classes based on whether each junctional read also has a genomic alignment. This seems like a quite inaccurate way of defining positive and negative training labels.

*-	We appreciate this comment on the reliability of the training reads for modeling true positive and false positives. We have now included two new Suppl. Figures 1 and 2 which show the distributions of the predictors used in the model in the simulated datasets (stratified by being true-/false-positive junctional reads (panel A) and positive/negative training read (panel B)). These histograms suggest that: 1- predictors that we have used in the model have predictive power as their distributions are separated in the reads aligned to the true positive and false positive junctions and 2- the distribution of the predictors in the negative/positive reads are similar to those based on the ground truth, suggesting the ability of our training selection criterion in capturing the general profile of TP/FP junctional reads. We have added this to the revised draft (Page 3, first paragraph):*

*“These predictors have power to distinguish reads aligned to false positive and true positive junctions defined by ground truth from simulated datasets. The positive and training reads used for training the model can reliably model the general profile of the true positive and false positive reads (Suppl. Figures 1,2). ”*

The lack of a genomic alignment for a putative junctional read does not mean it's a bona fide junctional read.

*-	Thank you for raising this concern about the definition of training data for the GLM. We do not mean to claim that any junction that does not have a genomic alignment is a bona fide junctional read. In fact, SICILIAN is meant to address this very problem: many of these alignments are *not* reliable due to sequencing artifacts. However, a prerequisite for any statistical regression model is to have an independent criterion for defining and selecting training data that can reliably represent the actual TP and FP calls. We do not say that any junction with a genomic alignment is an artifact and there might be still junctions with genomic alignment in our calls (providing that <10% of their reads align to a genomic region). Furthermore, we do not automatically call junctions without genomic reads as real and our main criterion for calling a junction is its SICILIAN score.*

Similarly, when evaluating SICILIAN using matched single-cell and bulk datasets, the authors defined junctions detected in the single-cell and bulk datasets of the same cell line as true positives. If I understand this correctly, this is basically using a subset of events detected by a given tool as the gold-standard to evaluate that tool.

*-	Thank you for clarifying this benchmarking analysis. Yes, this is correct and it is the basis of the evaluation of many papers (including a recent paper on the Sierra method published in Genome Biology (Patrick et al. 2020) for detecting different transcript usage in scRNA-Seq in which authors used the improved concordance between matched single cell and bulk data as a criterion for evaluating their method). We do not strictly define true positives as junctions found in both the bulk and single-cell datasets. We simply expect that junctions present in both the bulk and single cell datasets are more likely to be true positives, because both come from the same cell lines. Therefore, we measure concordance between the matched bulk and scRNA-Seq datasets before and after SICILIAN rather than defining specific ground truth. Again, this is a common approach for benchmarking in the absence of ground truth used in other single cell studies (such as Sierra (Patrick et al. 2020)).*

Finally, in the analysis of the lung data, the authors used annotated and unannotated junctions as surrogates for true positive and false positive junctions. This basically means any bona fide novel splice junction detected from the data will be automatically classified as a false positive.

*-	We appreciate the concerns raised about using annotation status as a proxy for ground truth. We do not mean to claim that all unannotated junctions are false positives. We simply use this well-accepted assumption in the field that annotated junctions are more likely to be true positives than unannotated junctions. This allows us to make an assessment of SICILIAN’s performance on real data by testing whether *in aggregate* SICILIAN maintains more annotated junctions than unannotated. Our method is unbiased and agnostic to annotation status and the annotation status is an orthogonal criterion we use for defining proxies for TP and FP junctions in real data. We understand that this is not the perfect definition but this is a well-practiced approach (used in other alignment papers such as (Patrick et al. 2020)) for defining FP and TP junctions in real data, where ground truth junctions are not available. We added more clarification that this is not a perfect proxy for ground truth in the text (Page 4):*

*“Annotation status is an approximate surrogate for ground truth as many junctions have been manually curated and or experimentally validated; we expect an algorithm that correctly identifies false positives to enrich for annotated junctions, particularly for organisms with extensive annotation (e.g., human)22”*

*Overall, the lack of consistency and rigor in defining gold-standards is a recurring issue in the manuscript that needs to be addressed.*

3. Read entropy seems like an important feature for the SICILIAN model, but there is no intuitive explanation for what it is in the Results section when the term is first introduced. There is a detailed explanation in Methods, but I think that comes too late.

*-	Thank you for pointing out the lack of sufficient explanation of entropy in the main text. We have now added an intuitive explanation on Page 3 and second paragraph, where entropy is first introduced (parts of the new explanation were moved from the Methods according to the suggestion):*

*“Read entropy, a quantitative measure of how repetitive a sequence is, is not generally appreciated as an important variable in scRNA-seq reads even though it is characteristic of technical artifacts, underlining its importance in the SICILIAN model. Read sequence entropy is expected to be a highly informative predictor of false-positive spliced alignments for two reasons: (1) reverse transcriptase or PCR enzymes are known to generate sequences of low entropy (“PCR stutter”), and PCR crossover is common in these regions; (2) these low-entropy sequences typically map to many places in the genome. Also, the entropy could be more informative and variable in scRNA-seq compared to bulk RNA-seq.”*

4.	A major novelty of SICILIAN is to statistically evaluate the confidence of the alignment for each junctional read, using multiple features of spliced alignment & read mapping. It would be very useful to assess which specific features make major contributions to the improved splice junction detection as reported for SICILIAN. The authors should consider dropping one feature at a time, re-train the model, and evaluate feature importance based on the drop in performance.

*-	We would like to thank the reviewer for the positive comment on the novelty of our approach. Since we have adopted GLMnet as the regression model, the coefficients for each predictor would shrink to 0, if it does not demonstrate predictive power for the data being processed. Therefore, by adopting GLMnet and using different predictors in our model, we provide an adaptive and highly-generalizable model for processing any type of scRNA-Seq data generated through different sequencing technologies. To further clarify this in the revised draft, we have added supplemental Figures 1 and 2 which show the separation between true and false positive training reads for each feature, which provides a way to visually assess the applicability of each feature for distinguishing between true and false positives and added the following to the text (Page 3):*

*“These predictors have power to distinguish reads aligned to false positive and true positive junctions defined by ground truth from simulated datasets. The positive and training reads used for training the model can reliably model the general profile of the true positive and false positive reads (Suppl. Figures 1,2).”*

5.	I do not understand the rationale for the multiple hypothesis testing correction in SICILIAN. The authors seem to think that splice junctions detected across multiple scRNA-seq samples are more reliable. While this certainly makes sense, implementing this as a filtering step means that condition-specific or individual-specific splice junctions would be filtered out, which does not seem like a good idea.

*-	We appreciate this concern about the method of multiple hypothesis testing correction, and the possibility that it could remove condition-specific splice junctions. Our multiple hypothesis testing correction uses the following rationale: if an aligner finds the same junction in two 10x lanes, we want to make one SICILIAN call for this junction that will hold across the whole individual, rather than call the junction as “real” in one lane and “not real” in another lane based on differing read features. In our analysis, we applied this postprocessing step to samples from the same individual rather than across all individuals at once, therefore we have not lost inter-individual heterogeneity in our splice calls. We should mention that the definition of the samples that can be considered for the postprocessing step in SICILIAN is quite flexible and as the first (Figure 1B) and second (Figure 1C) steps of SICILIAN are completely decoupled, users can decide which samples should be processed together in the postprocessing step, depending on the sample conditions and the scope of downstream analysis. We clarified this in the text (Page 7):*

*“To address this issue, SICILIAN adopts a multiple hypothesis testing correction strategy where for each junction, it collects the empirical p-values across the samples from an individual and then computes their median (or the “SICILIAN score”) as a unified criterion to decide whether the junction should be called across all 10x samples or SS2 cells (Figure 1B).”*

6.	SICILIAN was developed and tested entirely based on 10X datasets. To show the general applicability of this tool for splice junction detection from scRNA-seq data, the authors should demonstrate its use on data generated by other short-read protocols, such as Smart-seq2/3.

*-	Thank you for the suggestion to demonstrate the use of SICILIAN on technologies beyond 10x. Our model can easily be expanded to process Smart-seq2 data as well. SICILIAN’s model can automatically adapt to both single-end (i.e., 10X) and paired-end (i.e., SS2) data. When it processes PE data, it utilizes predictors from both R1 and R2 read alignments in the model and also extracts junctions from both R1 and R2 BAM files. We have now added the results based on running SICILIAN on ~6,500 Smart-Seq2 cells from the same two individuals from the human lung cell atlas (HLCA) study and updated Figure 2A and Supplementary Figure 5D to include results based on analyzing SS2 cells. We added the following explanation to the text on page 2:*

*“When running on paired-end data, SICILIAN utilizes alignment features for both mate reads (R1 and R2) in its model and extracts spliced junctions from both R1 and R2 alignments.”*

*We also added description to the Methods section to explain the predictors used in the model when input data is paired-end.*

7.	In Figure 1E, the improved performance of SICILIAN on the bulk-level simulated datasets seems too large to be true. The authors have already stated that SICILIAN is designed to address artifacts specific to scRNA-seq experiments, so why is there such a large increase in AUC value on bulk-level simulated datasets? Is there something wrong with the datasets or the evaluation? This also circles back to a key conceptual question about this work - what is the uniqueness of SICILIAN for scRNA-seq data as compared to bulk-level RNA-seq data?

*-	We would like to thank the reviewer for this comment. We have used two sets of well-established simulated data (HISAT and Engstrom) and the groundtruth for both datasets was provided by the authors of those datasets. We have also run STAR with default parameters and have provided the codes we used for benchmarking using these two datasets in SICILIAN the GitHub repository. We should mention that while the main purpose of developing SICILIAN was for splice detection in scRNA-Seq, many of the predictors that we have used in our model can be also used for distinguishing true positive and false positive in bulk data as well (as shown in Suppl. Figures 1 and 2). It is true that spliced aligners suffer from high false positive rates in both bulk and single-cell RNA-Seq and there is substantial room for improvement in splice detection. For example, in our previous work(Dehghannasiri et al. 2019), we showed that STAR-Fusion, a wrapper for fusion detection based on STAR chimeric alignments, finds a considerable number of gene fusions in the simulated dataset(Engström et al. 2013), which are all in fact false positive calls as the reads in this dataset are obtained based on modeling normal transcriptome and therefore the data is used as a negative control in benchmarking fusion detection algorithms.*

*To address the challenges of achieving high precision for junction detection across thousands of cells in a typical scRNA-Seq dataset, we specifically designed a second step by utilizing statistical evidence across all cells in the dataset to provide a unified criterion for each junction to be either called or discarded consistently across the entire scRNA-Seq dataset. Via this step, we achieved higher precision and provided a consistent calling procedure for each junction across the entire dataset which avoids creating biases for downstream analyses. Also, read entropy is one of the predictors in our model and as has been shown in Suppl. Figure 3, scRNA-Seq reads tend to have lower entropy values than bulk RNA-seq (the second paragraph on page 3):*

*“For example, in the 10x data from a human lung study, the average read entropy*

*in 20% of cells is < 4 (Supplementary Figure 3A), which is much more than the fraction of low-entropy reads in bulk RNA-seq datasets, e.g., the entropy is < 4 in only 0.09% and 0.4% of reads in one simulated and five bulk cell lines, respectively (Supplementary Figure 3B,C).”*

Minor issues:

1.	Page 2, Figure 1C appears out of order in the main text, before Figure 1A and 1B are referenced. This needs to be fixed.

*-	Thank you for pointing out this order discrepancy in the figures. We have now switched the order of the figure panels in Figure 1 so that they are referenced sequentially in the text.*

2.	Figure 1A, the diagram for "Junction-level statistical evidence collection" looks odd. None of the spliced alignments shown in the diagram appear to support the splice junction.

*-	Thank you for pointing this out. We have now modified the figure (now Figure 1B) in the revised manuscript.*

3.	Page 4, first paragraph: why do these numbers not add up to 100%? The description of results in this paragraph is somewhat confusing.

*-	It was very useful to us to hear that the wording of this paragraph was confusing. In fact, the numbers should not add up to 100%, because we are reporting numbers for annotated and unannotated junctions independently. Specifically, we are reporting the percent of called annotated junctions out of all annotated junctions, and the percent of called unannotated junctions out of all unannotated junctions. We have now modified this sentence to increase clarity (the second paragraph on Page 4):*

*“In all 10x datasets from four human and mouse individuals, SICILIAN calls a higher proportion of annotated junctions (83.6% of annotated junctions are called on average out of all annotated junctions) than unannotated junctions (29.2% of unannotated junctions are called on average out of all unannotated junctions), and a higher proportion of annotated junctional reads (87.6% of annotated junctional reads are called on average out of all annotated junctional reds) than unannotated junctional reads (23.9% of unannotated junctional reads are called on average out of all unannotated junctional reads), excluding junctions that only appear once in the dataset”*

4. Page 5, first paragraph: the authors discussed the potential application of SICILIAN to long-read RNA-seq data. This seems like a stretch and should be taken out, unless supported by data. The features and error profiles of long reads are very different from short reads.

*-	Thank you for suggesting this. We have now removed this statement, and the sentence now reads:*

*“The conceptual models used in SICILIAN are also applicable to other data types such as emerging single-cell sequencing technologies and bulk sequencing.”*

**Second round of review**

**Reviewer 1**

All comments in my previous review have been address satisfactorily.

**Reviewer 2**

The authors have addressed my comments well.
